# Investigation of dirigent like domains from bacterial genomes

**DOI:** 10.1186/s12859-022-04832-6

**Published:** 2022-08-02

**Authors:** Merlin Bardin, Pierre Rousselot-Pailley, Thierry Tron, Viviane Robert

**Affiliations:** CNRS, Centrale Marseille, iSm2, Aix Marseille Univ, Marseille, France

**Keywords:** Dirigent protein (DIRs), Stereoselectivity, Radical, Plant, Bacteria, Prokaryote, Bioinformatic analysis, Bacteria candidate, High product quality

## Abstract

**Background:**

DIRs are mysterious protein that have the ability to scavenge free radicals, which, are highly reactive with molecules in their vicinity. What is even more fascinating is that they carry out from these highly unstable species, a selective reaction (i.e., stereoenantioselective) from a well-defined substrate to give a very precise product. Unfortunately, to date, only three products have been demonstrated following studies on DIRs from the plant world, which until now was the kingdom where these proteins had been demonstrated. Within this kingdom, each DIR protein has its own type of substrate. The products identified to date, have on the other hand, a strong economic impact: in agriculture for example, the biosynthesis of (+)-gossypol could be highlighted (a repellent antifood produced by the cotton plant) by the DIRs of cotton. In forsythia plant species, it is the biosynthesis of (−)-pinoresinol, an intermediate leading to the synthesis of podophyllotoxine (a powerful anicancerous agent) which has been revealed. Recently, a clear path of study, potentially with strong impact, appeared by the hypothesis of the potential existence of protein DIR within the genomes of prokaryotes. The possibility of working with this type of organism is an undeniable advantage: since many sequenced genomes are available and the molecular tools are already developed. Even easier to implement and working on microbes, of less complex composition, offers many opportunities for laboratory studies. On the other hand, the diversity of their environment (e.g., soil, aquatic environments, extreme environmental conditions (pH, temperature, pressure) make them very diverse and varied subjects of study. Identifying new DIR proteins from bacteria means identifying new substrate or product molecules from these organisms. It is the promise of going further in understanding the mechanism of action of these proteins and this will most likely have a strong impact in the fields of agricultural, pharmaceutical and/or food chemistry.

**Results:**

Our goal is to obtain as much information as possible about these proteins to unlock the secrets of their exceptional functioning. Analyzes of structural and functional genomic data led to the identification of the Pfam PF03018 domain as characteristic of DIR proteins. This domain has been further identified in the sequence of bacterial proteins therefore named as DIR-like (DIRL). We have chosen a multidisciplinary bioinformatic approach centered on bacterial genome identification, gene expression and regulation signals, protein structures, and their molecular information content. The objective of this study was to perform a thorough bioinformatic analysis on these DIRLs to highlight any information leading to the selection of candidate bacteria for further cloning, purification, and characterization of bacterial DIRs.

**Conclusions:**

From studies of DIRL genes identification, primary structures, predictions of their secondary and tertiary structures, prediction of DIRL signals sequences, analysis of their gene organization and potential regulation, a list of primary bacterial candidates is proposed.

**Supplementary Information:**

The online version contains supplementary material available at 10.1186/s12859-022-04832-6.

## Introduction

The dependence on a protein extract for a stereo-control in the synthesis of lignans was observed for the first time in *Forsythia* species in 1992 [1]. The protein candidate was identified by Davin et al. in 1997 [2]. In this study, the Dirigent Protein (DIR) FiDIR1 from *Forsythia suspensa* was found as providing stereoselectivity in the coupling of radical oxidation products of E-coniferyl alcohol, leading to the exclusive formation of (−)-pinoresinol. Later, other DIRs involved in the formation of (+)-pinoresinol from different species [3, 4], (+)-gossypol from *Gossypium* species [5, 6] or (+) or (−)-medicarpin from *Glycyrrhiza echinata* and *Pisum Sativum* have been identified [7]. According to their relative homologies, DIRs were initially classified into different distinct height sub-families groups [8, 9]. The DIR-a family includes proteins involved in the stereoselective formation of (+) or (−)-pinoresinol. The DIR-b/d family includes proteins which have either a role in the synthesis of (+) and (−)-pterocarpan, or in the synthesis of diterpenoids such as the (+)-gossypol of cotton [6]. The DIR-c family is monocot specific; DIR domains are often fused to a jacalin and/or lectin domain [9]. The DIR-e are thought to be responsible for lignin deposition in the casparian strip localized at the endoderm level in primary roots [10]. The DIR-f could have a role in the defense of conifers against certain insects or to prevent injuries [8]. Proteins clustered in the DIR-g/h families have been so far poorly studied [11]. Globally, the DIRs from plant have a key role in secondary metabolite synthesis involved in defenses or attacks.

Recently, another classification distinguishing the “Lignans forming-DIR”, the “Terpenoids forming-DIR” and the “Pterocarpan forming-DIR” was proposed based on the three class of substrates DIRs are known to act on [12]. Pinoresinol forming DIR were the first to be studied and those for which a proposition for a mechanism is the most advanced [2, 13]. The initial radical forming reaction, *i. e.* the oxidation of coniferyl alcohol, is catalyzed by oxidative enzymes and is therefore not DIR dependent. In vitro, coupling coniferyl alcohol radicals results in a heterogenous mixture of dimeric compounds, *i.e.* (±) dehydroconiferyl alcohol, (±) pinoresinol and (±)-guaiacylglycerol 8-*O*-4’-coniferyl alcohol ethers. When a suitable DIR (*e.g., At*DIR6) is added to the reaction, one stereoisomer of pinoresinol is highly enriched. As DIRs have no radical forming activity on their own, in the absence of oxidase, no reaction will occur [2]. Therefore, DIRs are a class of proteins which dictate the stereochemistry of a compound, the synthesis of which, is initiated by other enzymes as oxidases.

**Fig. 1:**
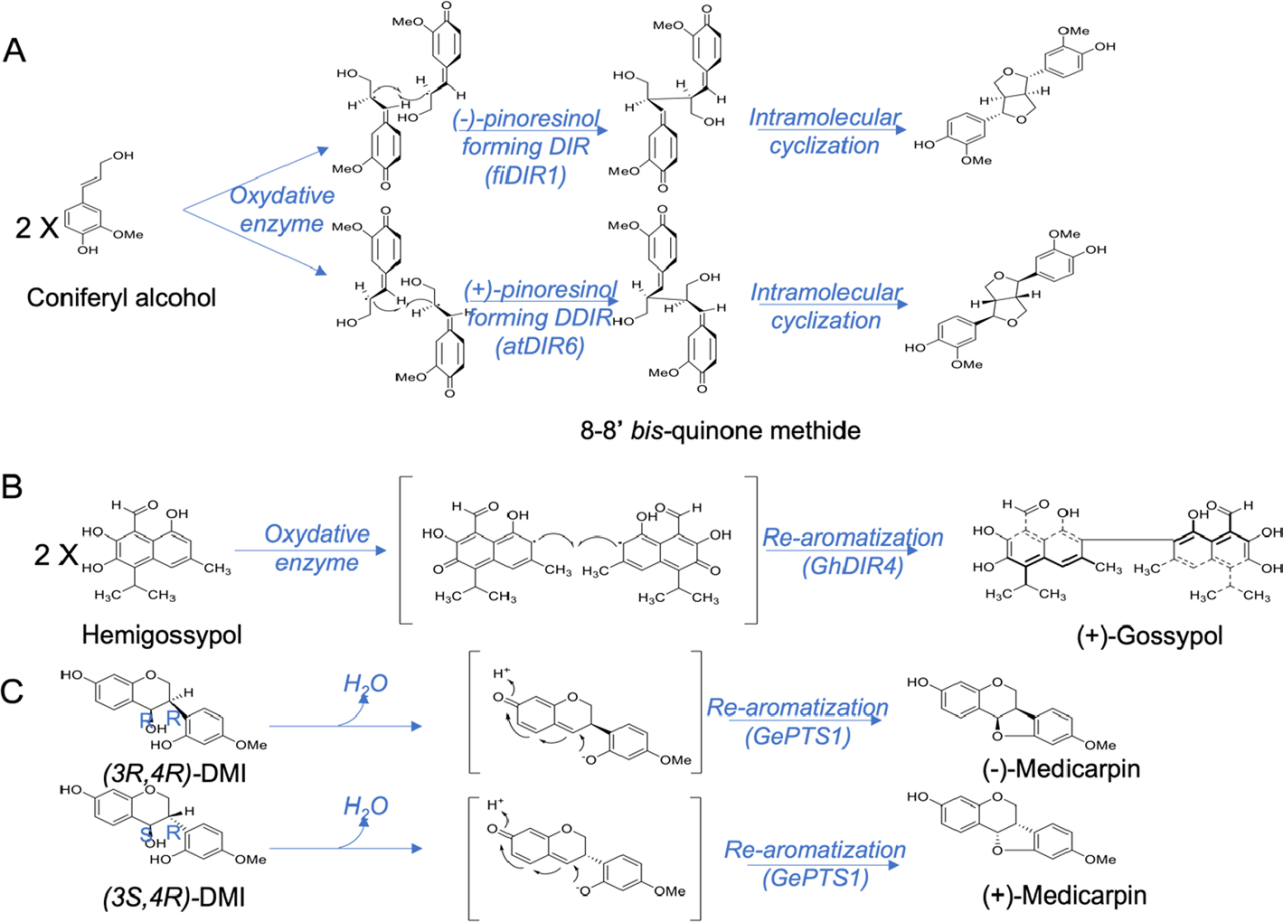
Mechanism of functioning of DIRs interacting with mono- or bis-quinone methides intermediates. **A** In lignans forming-DIR, Example of FiDIR1 for (1)-pinoresinol forming DP and atDIR6 for (+d)-pinoresinol forming DP. **B** In terpenoids forming-DIR GhDIR4 and **C** in Pterocarpan forming-DIR as GePTS1 (adapted from [10]). FiDIR1, Forsythia intermedia (−)-pinoresinol–forming DIR, AtDIR6, *A. thaliana* (+)-pinoresinol–forming DIR, GhDIR4, Gossipium.hirsutum-gossypol–forming DIR, GePTS1, Glycyrrhiza. echinata pterocarpan synthase 1

A common biochemical mechanism linking the binding and stabilization of distinct mono- and bis-quinone methide intermediates during different C–C and C–O bond–forming processes in plant has been suggested by Meng et al*.* in 2020 (Fig. 1, [14]). First, a mono-electronic oxidation generates a transient prochiral mono-quinone methide free radical. Depending on the nature of the substrate, this intermediate will then follow a specific pathway. Pinoresinol-forming DIRs promote either *si–si* or *re–re* coupling to afford chiral 8–8’-bis-quinone methides. This 8–8’-bis-quinone methides gives after intramolecular cyclization either (+)- or (–)-pinoresinol (Fig. 1A). (+)-gossypol–forming DIRs act on a prochiral free radical mono-quinone methide intermediate. It’s result from the mono-electronic oxidation of the achiral hemigossypol. The stereoselective coupling of the mono-quinone methide intermediate of hemigossypol gives a bis-quinone methide derivative. The re-aromatization of which generates the (+)-gossypol (Fig. 1B). The medicarpin-forming DIR also involves a mono-quinone methide generation and intramolecular cyclization/re-aromatization but from chiral isoflavonoids (3S,4R)-DMI and (3R,4R)-DMI substrates (Fig. 1C).

Beyond mechanistic considerations of radical coupling, very little information is available on how DIRs can interact with highly reactive radicals and orient the coupling. These studies are initially complicated by difficulties in identifying substrate-product pairs for each plant DIR candidate. More, like any study on plants, studies on plant DIRs are braked by the complexity of the plant kingdom: plants have complex nutritional and environmental needs, long periods of growth and limited molecular tools compared to laboratory microorganisms (as bacteria, fungi, or yeast). Identification of DIRs domains and their substrates in simpler organisms as prokaryotes could facilitates functional studies in the DIRs family. Secondary metabolites from bacteria are numerous. Microorganism as soil bacteria are known to allow the synthesis of many molecules of interest, such as antibiotics First, a mono-electronic oxidation generates [15]. The study of potential DIRs in prokaryotes could therefore shed new light on important molecules of pharmaceutical or industrial interest and on their biosynthetic pathway.

The massive sequencing of genomes in recent years provides an immense amount of data. Databases allow classification and automatic processing of all these information. In 2020, Dabravolski highlighted 42 bacterial proteins possessing a Dirigent Protein Like (DIRL) domain in Uniprot and Interpro databases [16]. Pursuing the study initiated on bacterial DIRL domains is important to gather more information in order to identify the most promising candidates for functional studies. Here, we performed a thorough bioinformatics analysis of DIRLs. Primary structures, predictions on secondary and tertiary structures, characterization of signals sequences, gene expression organization and regulation were compared to select bacteria candidates for further functional studies.

## Materials and methods

### Gene identification, characterization, alignment of the DIRL with *atDIR6* and phylogeny

**Fig. 2:**
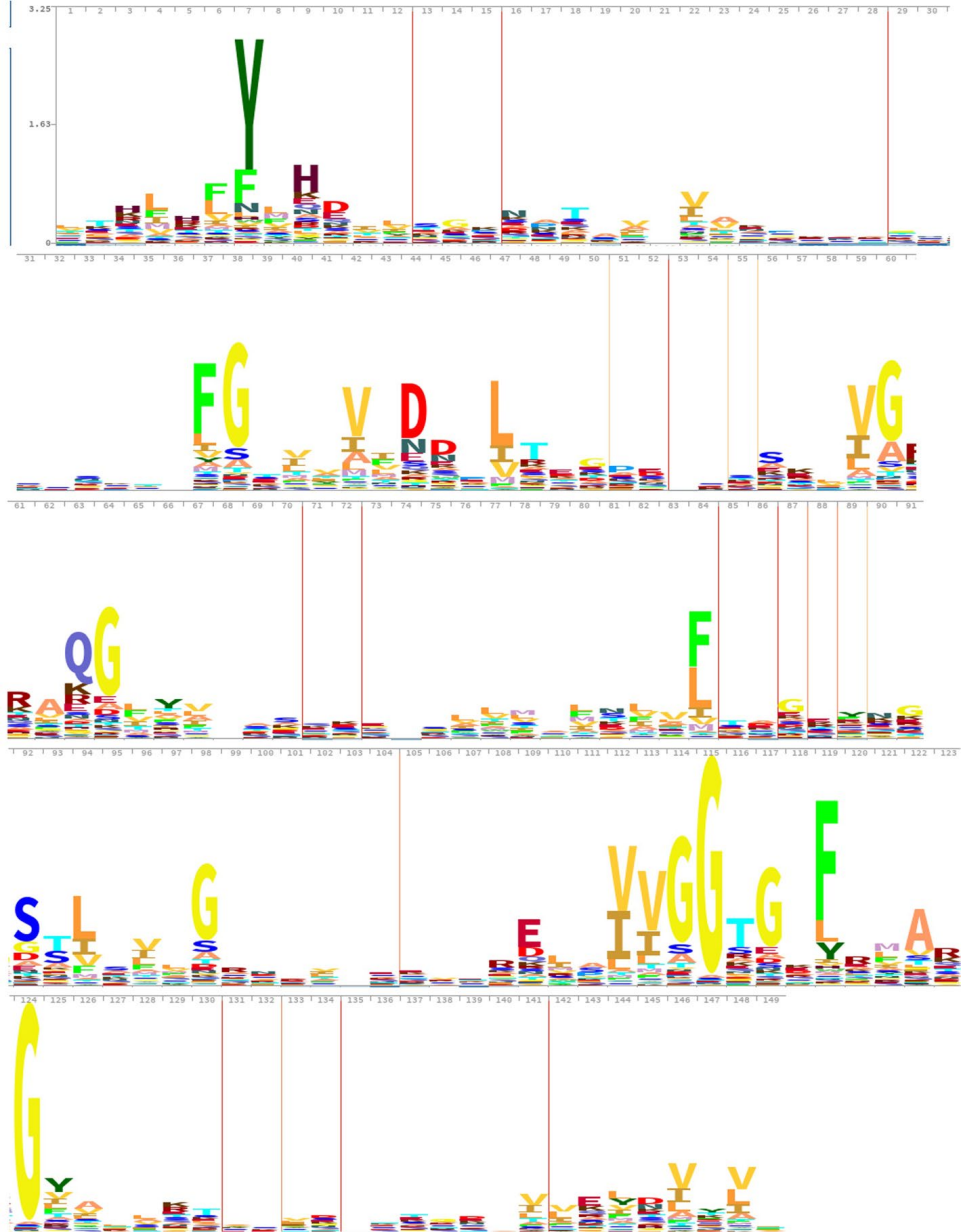
HMM Dirigent domain profile of Pfam PF03018 DIR family from available sequenced genomes

Interpro [17] was used for functional analysis of the updated list of DIRL proteins, for classifying them into families and predict domains and important sites. Like Pfam [18], Interpro uses the Hide Markov Model (HMM) to represent amino acids (AA) conserved in a profile, the one witch are bigger than the other (Fig. 2). One usually trains an HMM using an E-M algorithm. This consists of several iterations. Each iteration has one "estimate" and one "maximize" step. In the "maximize" step, each observation vector V is aligned with a state S in the model so that some likelihood measure is maximized.

Uniprot [19], was used to retrieve links to other databases (notably Ensembl, Interpro and the NCBI) and therefore information relating DIRLs and bacteria (Additional file 3: Table S1). Protein sequences in FASTA format were used to align sequences of DIRLs initially using Clustal Omega (ClustalO). Homology and identity (%) between bacterial DIRLs and plant DIRs were retrieved from global alignments with ClustalW (Table 1, column 6 and 7). AtDIR6 was chosen as reference in the comparison to the DIRLs of bacteria.

**Table 1:** Summary of information on the 49 bacteria having a DIRL protein

n°	Bacteria species	Coloration of Gram	UniprotAccession number of DIRL	Secondary metabolism clusters via AntiSMASH version 6.0	Protein size (AA)	Identity % with AtDIR6 (clustalW) (%)	Similarity % with AtDIR6 (%)	Peptide signal prediction size (AA) by The SignalP 5.0 tool	S–S bridge prediction position/probability Score by AlphaFold	N-Glycosylation prediction in prokaryotes via Glycopp with score > 0,8	Bacterial family number via phylogenie results
1	*Streptomyces sp. Root1304*	+	A0A0T1SRD5	23	166	22	30	29	84–98	0	I
2	*Streptomyces sp. PanSC19*	+	A0A3N1Q171	22	167	22	27	30	85–99	0	I
3	*Streptomyces sp. 62*	+	A0A3D9LXQ3	29	167	22	33	30	85–99	0	i
***4***	***Sphaerobacter thermophilus strain DSM 20745***	***+***	***D1C1V3***	***1***	***173***	***21***	***35***	***36***	***155–169***	***0***	***NO***
5	*Kutzneria albida DSM 43870*	+	W5WKJ3	46	164	21	28	34	no S–S bridge	0	NO
6	*Streptomyces vietnamensis GIMV4.0001*	+	A0A0B5I9B2	28	160	20	29	32	87–101	**0**	i
7	*Streptomyces viridochromogenes*	+	A0A0L8K704	0	166	20	27	29	84–98	0	i
8	*Cystobacter fuscus DSM 52655*	−	A0A250IWY0	47	154	20	39	27	81–92	**0**	IV
9	*Streptomyces bottropensis ATCC 25435*	+	M3F484	30	161	19	30	29	89–100	0	NO
***10***	***Nocardioides iriomotensis NBRC 105384***	***+***	***A0A4Q5J010***	***5***	***152***	***19***	***32***	***24***	***91–92***	***0***	***NO***
11	*Methylomicrobium alcaliphilum*	−	G4T3L1	9	147	19	31	19	76–87	**0**	iii
12	*Archangium violaceum Cb vi76*	−	A0A084SGS8	0	148	19	32	21	75–86	**0**	IV
13	*Chloroflexi bacterium GWC2_73_18*	−	A0A1F8LMI0	1	155	18	31	25	82–93	0	NO
14	*Nocardia mexicana DSM 44952*	+	A0A370GIR3	38	161	18	29	35	87–101	0	NO
15	*Gammaproteobacteria bacterium HGW*	−	A0A2N1ZX47	10	146	18	30	18	75–86	0	III
16	*Cystobacter fuscus DSM 2262*	−	S9NWQ1	44	137	18	27	ND	64–75	**0**	IV
17	*Archangium gephyra*	−	A0A0L8K704	41	151	17	27	19	78–89	**0**	IV
18	*Cystobacter ferrugineus str. Cbfe23*	−	A0A1L9B5J6	41	137	17	27	ND	64–75	**0**	IV
19	*Streptomyces sp. SA15*	+	A0A2A2YWR6	51	206	17	33	30	106–118	0	V
20	*Stigmatella erecta*	−	A0A1I0JK20	29	162	17	25	ND	89–100	**0**	IV
21	*Streptomyces alboflavus str. MDJK44*	+	A0A1Z1WCN3	29	163	17	32	27	no S–S bridge	0	II
22	*Streptomyces sp. NWU339*	+	A0A2U2Z012	32	205	17	32	30	105–112	**0**	V
23	*Streptomyces formicae KY5*	+	A0A291Q4S1	45	163	16	28	33	no S–S bridge	0	II
24	*Streptomyces sp. CB02400*	+	A0A1Q5L121	33	205	16	28	30	105–117	0	V
25	*Streptomyces caelestis*	+	A0A0M8QLY2	42	205	16	27	30	105–117	0	V
26	*Pseudonocardiales bacterium YIM PH 21723*	+	A0A2W6DT35	4	168	16	28	33	96–109	0	NO
***27***	***Methylomicrobium kenyense (AMO1)***	***−***	***A0A543V9V5***	***7***	***149***	***16***	***27***	***ND***	***2 S–S bridges:76–148/87/123***	***0***	***III***
28	*Streptomyces griseoruber str. DSM 40281*	+	A0A101SV50	36	161	16	25	29	89–100	**0**	NO
29	*Chloroflexi bacterium*	−	A0A3A0ARB1	9	201	16	27	27	only one Cys	0	NO
***30***	***Thiogranum longum str. DSM 19610***	***−***	***A0A4R1H9Y2***	***3***	***148***	***16***	***25***	***21***	***76–87***	***0***	***III***
31	*Streptomyces fungicidicus str. TXX3120*	+	A0A494UTW6	17	205	15	28	ND	104–116	0	V
32	*Streptomyces toyocaensis str. NRRL 15009*	+	A0A081XHD4	28	205	15	29	30	105–117	**0**	V
33	*Streptomyces showdoensis ATCC 15227*	+	A0A2P2GJ53	29	167	15	21	29	86–99	**0**	I
34	*Enterobacter ludwigii str. P101*	−	W0BV16	6	154	15	22	19	83–94	0	NO
35	*Stigmatella aurantiaca*	−	A0A1H7HD66	28	162	15	25	35	89–100	**0**	IV
36	*Modestobacter sp. DSM 44400*	+	A0A1H3KGG2	5	123	15	25	ND	41–52	0	NO
37	*Streptomyces radiopugnans*	+	A0A1H9DCJ1	2	205	15	27	32	105–117	0	V
***38***	***Methylomicrobium buryatense* str. 5GB1C**	***−***	***A0A4P9UY52***	***9***	***131***	***15***	***26***	***ND***	***no S–S bridge***	***0***	***III***
39	*Vitiosangium sp. GDMCC 1.1324*	−	A0A2T4VJB7	56	146	15	25	ND	74–85	**0**	IV
40	*Archangium sp. Cb G35*	−	A0A1Q3H6L3	48	145	15	23	ND	72–83	**0**	IV
***41***	***Cellulomonas aerilata***	***+***	***A0A512DF30***	***3***	***174***	***14***	***25***	***24***	***99–115***	***0***	***NO***
42	*Streptomyces sp. NRRL S-4*	+	A0A0N1K8E7	43	203	14	25	30	105–117	0	V
43	*Streptomyces silvensis ATCC 53525*	+	A0A0W7X1T4	**50**	166	14	26	33	no S–S bridge	0	II
44	*Streptomyces sp. CNZ306*	+	A0A2M9A2V5	7	258	14	27	ND	158–170	0	V
45	*Streptomyces dysideae sp. RV15*	+	A0A124IFP4	32	187	13	23	36	112–124	0	V
46	*Streptomyces regalis*	+	A0A0X3UWY4	41	122	13	18	ND	47–59	0	V
47	*Streptomyces sp. 13–12-16*	+	A0A1X4I170	42	205	12	24	30	105–117	0	V
48	*Streptoalloteichus hindustanus*	+	A0A1M5GN54	31	161	12	22	31	87–101	0	V
49	*Hyalangium minutum*	−	A0A085VSV8	34	96	12	20	ND	no S–S bridge	**0**	IV

2021 additions to the list published by Dabravolski in 2020 are highlighted in bold. The DIRLs belonging to a predicted operon structure via AntiSmash logiciel are the 15 bacteria underlined

**Fig. 3:**
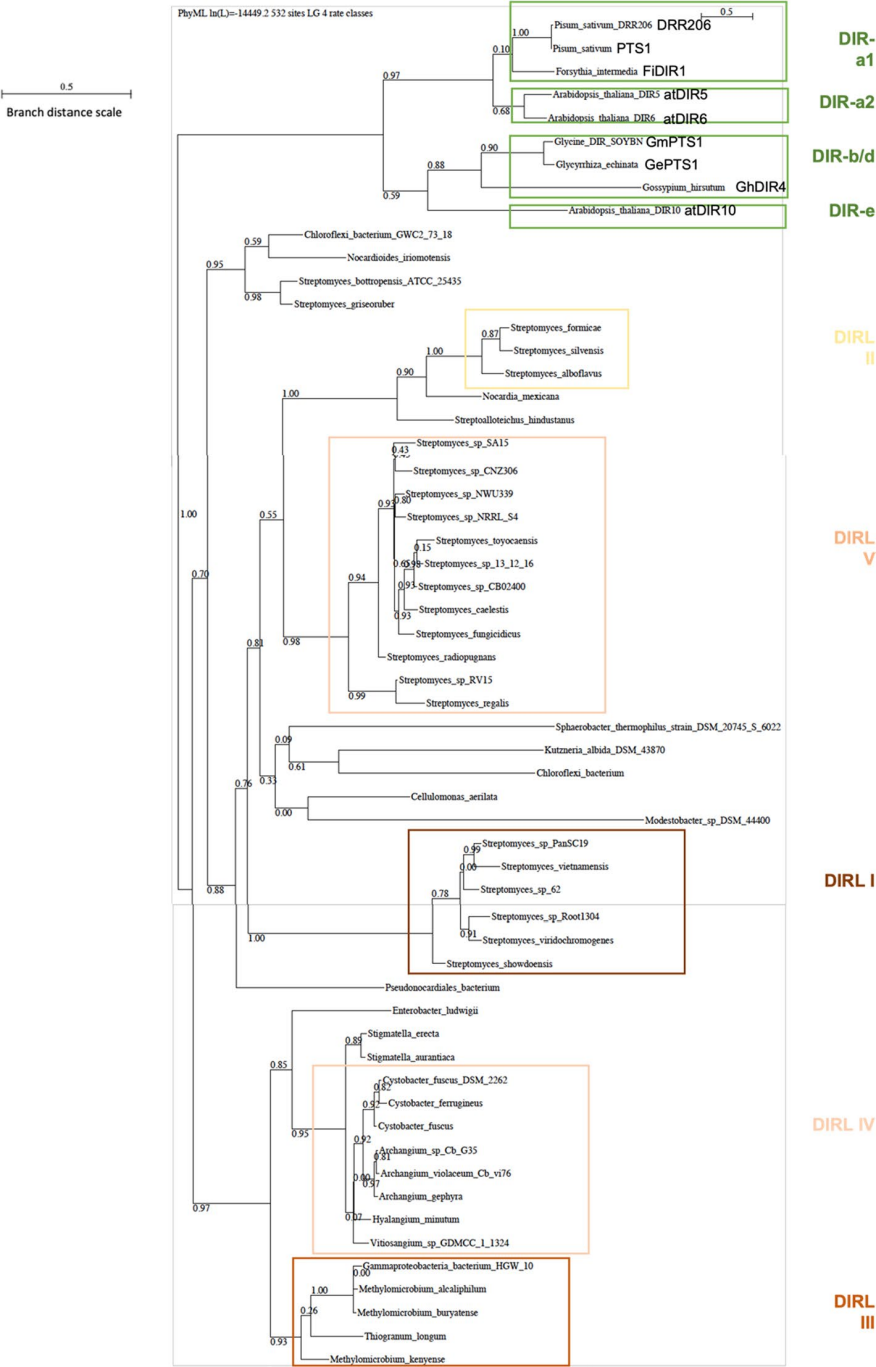
Phylogenetic tree constructed by Seaview server according to the Neighbor-Joining method, corrected by ML method. In green, branches linked to the 8 best characterized plant DIRs (PTS1 and DRR206 from *Pisum sativum* and FiDIR1 from fosythia intermedia are from family DIR-a1. AtDIR5 and atDIR6 from *Arabidopsis thaliana* are from family DIR-a2. GmDRR1 from Glycine max, GePTS1 from Glycyrrhiza echinanta and Gossipium Hirsitum GhDIR4 are from family DIR-b/d. AtDIR10 from *A. thaliana* is from family DIR-e); in black, branches connecting the 49 bacterial DIRLs; groups of similar DIRLs are squared and numbered in 5 groups DIRL I to IV. Branch Distance scale is indicated. Bacteria which do not belong to family I to V and not clustered are not squared

Phylogeny was analyzed using the Seaview software version 5.0.5 [20]. From protein sequences in FASTA format aligned via ClustalO, the neighbor-joining agglomerative method was used and corrected by ML method (Maximum Likehood method) to create the phylogenetic tree of Fig. 3 and the sequence alignment of Additional file 1: Fig. S1. *F. intermedia* FiDIR1, *P. sativum* DRR206, *G. echinata* PTS1, *A. Thaliana* atDIR5 and *A. Thaliana* atDIR6 were selected among plant sequences (the 3D structures of AtDIR6, DRR206 and PTS1 are resolved and FiDIR1 and AtDIR5 are also well characterized) (Additional file 1: Fig. S1).

### Primary, second and tertiary structural analysis

The SignalP 5.0 tool (https://services.healthtech.dtu.dk/service.php?SignalP-5.0) was used for the prediction of signal peptides in bacterial DIRLs (Table 1, column 9 and confirmed by Alphafold).

The sequences were entered in fasta format for Glycopp server, PredictProtein and Alphafold2.

Glycopp server [21] was used for the prediction of N- and O Glycosites in prokaryotic protein sequences (Table 1, column 11).

PredictProtein [22] was used to predict the secondary structure of DIRLs. β strands, interloops and signal peptides identified via PredictProtein are consistent with results from other prediction softwares as well as with the structure of AtDIR6 (data not shown).

Alphafold2 [23] (https://colab.research.google.com/github/sokrypton/ColabFold/blob/main/beta/AlphaFold2_advanced.ipynb) was used to produce 3D models of bacterial DIRLs (Fig. 5 for DIRL representing DIRL famillies).

TM-Align [24] is an algorithm for protein structure alignment and comparison based on statistics. It allows to process a 3D visualization of the structural alignment. The RMSD (Root-Mean-Square Deviation) is the measure of the average distance between the atoms (usually the backbone atoms) of superimposed proteins. The pdb prediction structures from DIRs and DIRL protein is compare with the one from atDIR6 (LAL5) (Additional file 3: Table S1, column 5). This software calculs also the alignment length (Additional file 3: Table S1, column 3) and the Seq_ID (number of residues witch are identical / the number of the residues aligned) (Additional file 3: Table S1, column 5).

### Genomic analysis of the DIRL region

Bacterial genomes from Table 1, column 2 were all recovered from NCBI (https://www.ncbi.nlm.nih.gov). Ensembl/EMBL (European Molecular Biology Laboratory, http://www.ensembl.org) was used for genomic analysis.

SoftBerry (http://www.softberry.com) enables comparison of genomic structures or sequences and was used for the prediction of promoters in areas upstream of DIRLs via the BPROM program (BPROM: Bacterial sigma 70 promoter prediction program).

AntiSMASH version 6.0 [25] was used for the analysis of bacterial genomes (Table 1, column 2) to identify gene clusters involved in the biosynthesis of secondary metabolic compounds Table 1, column 5). Once listed and located within the genomes, clusters were analyzed to check for the presence of a DIRL locus within a cluster.

To go further, the location of DIRL genes in genomes was also used to identify potential operonic structures. There are several cumulative methods for identifying operonic structure in bacteria. The first is taking into consideration the orientation of the genes as well as the intergenic space. If genes are in the same orientation and with a short intergenic space (or even overlapping) it is probable that these genes belong to the same operon. The second method rely on the identification of genes in the target area that may have a role in the structure of an operon. Here, these genes are often transcriptional regulators, transmembrane transporters, phosphatases or even kinases. The third method is based on the identification of promoters. An operon requires few promoters if not a single promoter. Each genomic region containing a DIRL locus was therefore analyzed with a magnifying glass.

The SoftBerry software was used to identify forward sequences binding the sigma 70 promoter, characteristic of bacteria. If regions where the DIRL loci are located contain a low number of sigma 70 promoters and if such a promoter localizes upstream of an "operonic structure", then this is in favor of an operon.

## Results

### Dirigent domain and data bases analyses

The Pfam database contains an HMM profile specific to the Dirigent Domain: Pfam PF03018 (Fig. 2). This profile highlights very conserved positions within the family of DIRs. The Interpro database reference two families comprising genes encoding proteins exhibiting either a “Dirigent protein” domain (IPR004265), or an “Allene oxide cyclase/Dirigent protein” domain (AOC/DIR IPR044859). It should be noted that in plants, the dirigent domain of DIRs is structurally close to the domain of allene oxide cyclases [26]. These informations have been previously used by Dabravolski in 2020 to highlight the existence of genes with a putative dirigent domain in bacterial genomes [16].

At the start of our study, we updated Dabravolski's list of bacterial candidates considering data newly deposited in Interpro (between April 2020 and October 2021). Today, the "Dirigent domain" and "AOC/DIR" families contain respectively 9000 and 10,000 genes, the vast majority of which are from plant genomes. There are also some other eukaryotes: Insects, Micro-seaweed, fungi, and yeast. To the 42 bacterial genomes in which Dabravolski initially revealed the presence of DIRL domains we included in our study 7 new bacterial genomes containing DIRL encoding sequences for a total of 49 bacteria possessing a gene encoding a DIRL (Table 1, column 2). In plant, as for many functional genes, the number of DIRs encoding genes is high (*e.g.,* 25 genes in *Arabidopsis thaliana*). To the contrary of plants, the 49 bacterial genomes examined contain a single gene encoding a DIRL per genome. It should be also noted that if, for reasons not yet determined, some plant DIRs are made of several dirigent domains, the DIRLs considered in this study only have a single domain rarely exceeding 200 amino acids, comparable in size to single-domain plant DIRs (Table 1, column 6).

Among bacteria possessing a gene encoding a DIRL most are from the phylum Actinobacteria and particularly from the genus *Streptomycetaceae*. Other Actinobacteria are *Pseudonocardiaceae*. *Archangiaceae* from the phylum Deltaproteobacteria represent 20% of the total. The 12% of Gammaproteobacteria are mainly from the *Methylococcaceae* genus. Three bacteria are *Chloroflexi*. Among the 49 bacteria, *Actinobacteria* are gram positive, the other phyla are gram negative (Table 1, column 3). Most of the bacteria carrying a DIRL encoding gene have been isolated from soil or aquatic environments such as *Methylomicrobium alcaliphilum* found in a saline lake in Asia. Some mycorrhizal or symbiotic bacteria are also identified. As *Streptomyces formicae*, found associated with a fungus in the heads of ants in China and Africa. As *Streptomyces sp. Root1304* found in the roots of *A. thaliana*. Interestingly, many of these bacteria have been identified as synthesizing molecules of interest, including antibiotics [27]. For example, bottromycin produced by *Streptomyces bottropensis* inhibits the resistance of certain bacteria to other antibiotics [28]*.* Formicamycin synthesized by *S. formicae* is effective against *Staphylococcus aureus* methicillin-resistant (MRSA) and vancomycin-resistant Enterococci (Vancomycin-RE) [29]. Also present in this group, *Streptomyces viridochromogenes* synthesizes the optically active herbicide bialaphos [30].

### Sequences alignment analysis

The identity and similarity percentages shown in Table 1 columns 7 and 8 are the result of an overall alignment performed with ClustalW using *At*DIR6 protein as query and the fasta sequences of DIRL from Bacteria. The percentage of identity varies between 22 and 12% for an average of 17%. The percentage of similarity is comprised between 39 and 18% with an average of 27%. These values are similar to those found among plant DIRs (30% between PTS1 and *At*DIR6 for example) [14].

A phylogenetic tree grouping together the 49 sequences of bacterial DIRLs and 8 sequences of selected plant DIRs (based on published representative members from plant families) was constructed. The tree shown Fig. 3 allows a clear distinction of 5 groups among DIRLs: *Streptomycetaceae* are spread in groups I, II and V, group IV contain *Deltaproteobacteria,* and group III contains *Gammaproteobacteria* (Table 1, column 12). For the branch grouping *Nocardioides iriomotensis* NBRC105384, *Pseudonocardiales* bacterium YIMPH 21723, *Chloroflexi* bacterium GWC27318, *Streptomyces bottropensis* ATCC25435 and *Streptomyces griseoruber* DSM 40281, distances were here evaluated as too long to consider it as a 6^th^ group. On the other hand, sequences annotated as allene oxide cyclases cluster are on this branch.

DIRL sequences were aligned with a selection of five best characterized sequences of plant DIRs (Additional file 1: Fig. S1): *F. intermedia* FiDIR1, *P. sativum* DRR206, *G. echinata* PTS1, *A. Thaliana* atDIR5 and *A. Thaliana* atDIR6 (3D structures of AtDIR6, DRR206 and PTS1 are resolved and FiDIR1 and AtDIR5 are also well characterized). Plant DIRs are mainly organized in a β -barrel made of up to 8 β strands [13, 24]. A similar structural organization is proposed for the DIRLs. From an analysis with the PredictProtein software, DIRLs would have a number of strands comprised between 4 and 8.

Plant DIRs genes generally encode a signal peptide that allows either the anchoring of the DIR in the plasma membrane or their secretion into the apoplasm. SignalP server was used to check for the presence or not of a signal peptide in DIRLs from bacteria. Three types of signal peptides are found in Archaea and bacteria: 1—Sec/SPI: standard secretion signal peptide transposed by the Sec translocon and cut by signal peptidase I; 2—Sec/SPII: transposed signal peptide also by the Sec transposon but cut by signal peptidase II and 3—Tat/SPI: signal peptide Tat transposed by the Tat translocon and cut by signal peptidase I).

From this search, only 12 DIRLs on the 49 sequences considered would not possess an identifiable signal peptide, 9 of which being among the shortest sequences of the panel (i*.e.,* < 150 residues) and one (*Streptomyces* sp. CNZ306, 258 residues) being the longest sequence (Table 1, column 9). Among the 38 DIRL with a potential signal sequence only that from *Kutzneria albida* DSM44400 contains a predicted Tat signal (data not shown). The presence of a Tat signal suggests that the corresponding protein is excreted as a folded protein, unlike protein sequences containing a Sec signal which can be secreted as non-folded proteins. Potential non-cytoplasmic DIRLs could be either membrane anchored or released into the extracellular medium.

Protein glycosylation is an important post-translational modification process in eukaryotic proteins. In plant DIRs, glycosylation is essential for the activity [3]. Prokariotic proteins, in particular secreted ones, can also be glycosylated [31]. We checked the presence of potential glycosylation sites in DIRLs as these proteins could be secreted in several bacteria and as most DIRLs contain Aspartate residu on their sequences. From our analysis using Glycopp, a server specialized to glycosite prediction in prokaryotes, we cannot conclude on the presence of glycosylation sites in DIRLs (Table 1, column 11).

Pinoresinol forming DIRs have a disulfide bridge connecting a cysteine at the C-terminus to a cysteine at the N-terminus of the proteins (in purple on Additional file 1: Fig. S1). This bridge has been proposed for stabilizing the 3D barrel structure [26]. Not present in pterocarpan and gossypol forming DIRs, it seems that this structural element is also not present in DIRLs at least in this area. In fact, besides cysteines found in potential signal peptides, two remarkably conserved cysteines are located in the consecutive β3 and β4 strands in 42 of the 49 DIRLs. These cysteines could pair as a disulfide bridge as it is revealed by Alphafold2 prediction (Table 1, column 10). Yet, the function of such a structural element rigidifying consecutive and adjacent strands is difficult to envision. Note that in plant DIRs this region is thought to be part of the substrate binding pockets” (i.e., the cavity where radicals could bind [24, 30–32]. In addition to this potential disulfide bridge connecting β3 and β4 strands, one sequence (*Methylomicrobium kenyense*) contains two other cysteine residues potentially pairing loop VIII (connecting the β7 and β8 strands) and the penultimate C-terminal position (Additional file 1: Fig S1).

**Fig. 4:**
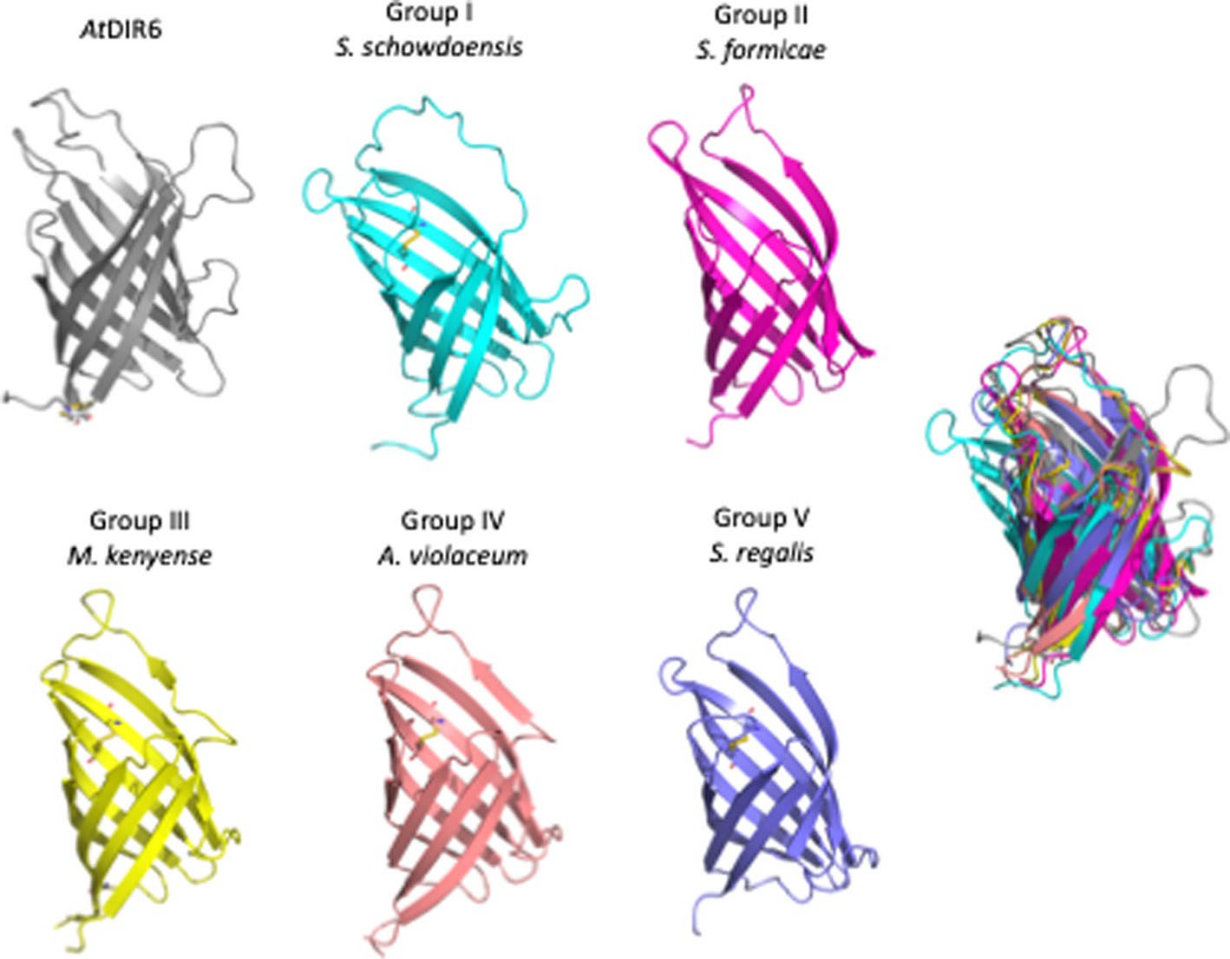
3D model of AtDIR6 (5LAL) and 3D model predictions of DPLs representative of family I to V. A superposition of the 6 models is presented far right

3D models were constructed via Alphafold2 [23] initially using full-length sequences in fasta format of DIRLs as input (*i. e*. from which the predicted signal sequences were not deleted) and the fasta sequence of atDIR6. The structural proximity of the different DIR and DIRLs proteins was studied using the RSMD calculation the 3D prediction of each DIRLs (Additional file 3: Table S1, column 4). The RSMD distance between atDIR6 and DIRLs is not superior to 3,68 Å compared to 3 Å between atDIR6 and atDIR10 3D structures (the post important RMSD distance between the DIR presented in Additional file 3: Table S1). In all the models obtained, DIRLs monomer fold around a β barrel core with a large cavity open opposite to the N- and C-termini. Models are overall very similar to known plant DIR monomers 3D structures. In all the models of the 36 DIRLs for which a signal sequence was predicted from our SignalP runs, the N-terminal sequence appears unfolded almost up to the beginning of the β1 strand. This strongly supports the existence of a signal peptide for all DIRLs. A selection of models of DIRL representative of each branch of the tree is shown Fig. 4. The remarkably well conserved region spanning 13 residues (A/P-GGTG-Y/F—S/RG) at the end of the β6 and the beginning of the loop VII mentioned earlier (Additional file 1: Fig. S1) would be partly forming the inter-monomers wall of a putative trimeric structure. This sequence might be important for the oligomerization of the peptide chain.

### Genomics and operon research

**Fig. 5:**
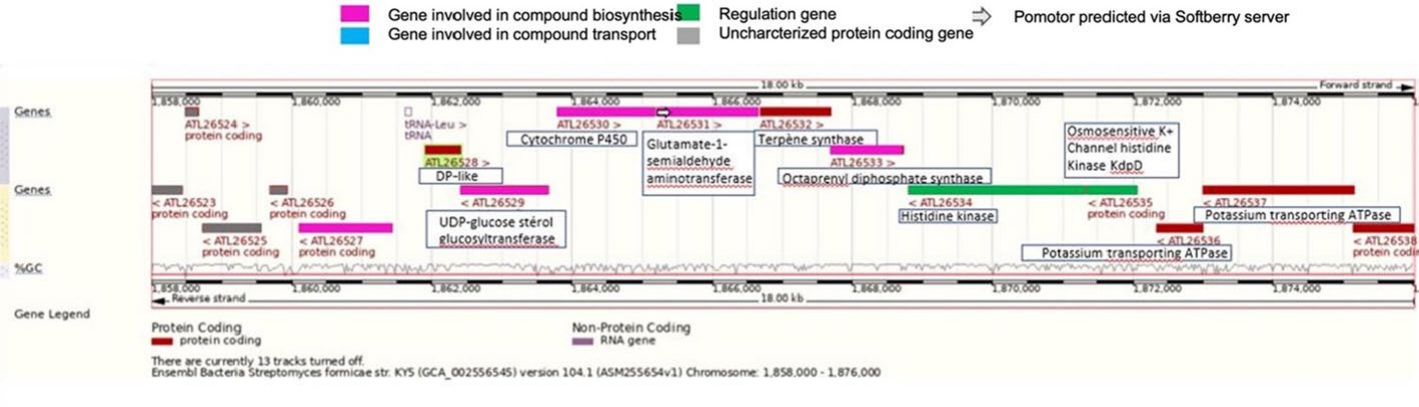
Schematic representation of a part the genome of Streptomyces formicae, annotated manually in order to obtain genomic information around the genes encoding a potential DIRL

Bacteria highlighted in this study mainly come from the soil and are reputed to have a high secondary metabolism with potential for producing bioactive molecules. The AntiSMASH tool [25] was used to analyze each genome with the goal to identify genes and their clusters related to biosynthetic pathways. AntiSMASH predicted several clusters (Table 1, column 5) (from 1 up to 56 different (for *Vitiosangium* sp. GDMCC 1.1324, Table 1 lane 39), for each of the analyzed genome with the notable exceptions of the *Archangium violaceum Cb vi76* and *Streptomyces viridochromogenes* genomes in which no cluster was predicted (Table 1, lane 12 and lane 7 respectively). By crossing the data with those of Table 1 column 5, looking for the presence of DIRLs coding gene within these clusters, 2 bacteria, *Streptomyces silvensis* and *S. formicae* were found to have a cluster including a DIRL gene. The cluster identified in the region 15 of *S. formicae* is thought to contains potential terpen and octaprenyl synthases (see below Fig. 5). The biosynthetic pathway corresponding to the cluster found in the genome of *S. silvensis* is unknown yet. This survey revealed DIRL genes are probably embedded in an operonic structure in 10 bacterial genomes (species underlined in Table 1).

The 47 DIRL genes which were not associated to a cluster of genes encoding for known secondary metabolism biosynthetic pathways revealed via AntiSmash are however still interesting from a genomic point of view. Information on the genomic organization around DIRL genes was collected via Uniprot and “Ensemble bacteria” servers. Genes close to the DIRL locus as well as their orientation and their genomic organization were therefore studied for each of the bacteria in the study (*e.g*., *S. formicae*, Fig. 5 and Additional file 2: Fig S2 for some interested others). Several genomes share similarities in their genomic structure around DIRL genes: in few bacteria, the closest or the two closest genes are in reverse orientation; in other bacteria, the closest or the two closest genes have the same orientation (Additional file 2: Fig S2). DIRLs genes are, in most cases, adjacent to genes encoding enzymes or proteins not yet characterized. On the other hand, for some bacteria, the DIRL gene is located next to a gene encoding a hydrolase or an oxidoreductase. Moreover, some DIRLs genes are close to a gene encoding a secreted protein of unknown function as in *Stigmatella aurantiaca* and *Stigmatella erecta*, known to secrete antibiotics. Note that for 24 genomes this analysis was not possible since the chromosomic regions were not available (e.g., end of the contig or the genome is not complete yet).

To go further, the location of DIRL genes in genomes was also used to identify potential operonic structures. There are several cumulative methods of identifying operonic structure in bacteria (see Material and Method). Each genomic region containing a DIRL locus was therefore analyzed with a magnifying glass. The SoftBerry software was used to identify forward sequences binding the sigma 70 promoter, characteristic of bacteria. If regions where the DIRL loci are located contain a low number of sigma 70 promoters and if such a promoter localizes upstream of an "operonic structure", then this is in favor of an operon. This survey revealed DIRL genes probably embedded in an operonic structure in 10 bacterial genomes (species underlined in Table 1, Additional file 2: Fig. S2).

## Discussion

In this study we focalized on the identification of DIRLs candidates in bacteria from the many sequenced bacterial genomes available. The structural and functional genomic analyzes centered on the DIR domain Pfam PF03018 presented in this work allowed us to confirm the work of Dabravolski [16] and to identify 7 new bacteria containing a gene encoding a DIRL.

Bacteria possessing a gene encoding a DIRL are mainly saprophyte and mesophilic, rarely pathogen. Most of them are soil bacteria. Few are extremophile as *Sphaerobacter thermophilus* strain DSM 20745, *Streptomyces alboflavus str.* MDJK44 or *Thiogranum longum str*. DSM 19610 which was isolated from a deep-water hydrothermal spring with high salt concentration [33]. Some were isolated from lake or sea waters as *C. bacterium* which is a bacterium from a Siberian soda lake described to be anaerobic and photosynthetic [34]. Six were isolated from forest or from habitats or to be linked with plants*, e.g*., *Enterobacter ludwigii* str. P101 that is an endophyte from plant pea or *Streptomyces sp.* Root1304 that is present in the microbiote of *A. thaliana* roots. Out of the 49 bacteria studied, 31% are probably able to synthesize antibiotics but no link was obtained between DIRLs and biosynthetic antibiotics pathways in this study.

Our analysis of DIRLs sequences led to predictions that are therefore subject to discussion. However, most of the sources overlapped and led us to similar predictions. Analysis of primary structures, predictions of secondary and tertiary structures, characterization of DIRL signal sequences and the study of gene regulation and locus localization and organization has enriched our knowledge on DIRLs. Presently, among the 49 DIRLs identified some criteria can be retained for the selection of a DIRL candidate to be studied.

Most of DIRLs should be either membrane anchored or secreted since most of the DIRLs coding gene contain a predicted N-terminal sequence addressing the sequence to the Sec or Tat membrane pathways. In the alignment presented Additional file 1: Fig. S1, residues conserved in DIRs and in DIRLs or in both protein groups are highlighted. Some of these AAs are also found in the HMM profile presented above Fig. 1. The majority of AA conserved in all species are located between the β2 and β7 strands. The most remarkably conserved AAs between the DIRs of plants and DIRLs bacteria are glycine residues. Glycine are small residues which provide flexibility to the protein structures. A region spanning 13 residues (A/P-GGTG-Y/F—S/RG) is remarkably well conserved between the end of the β6 and the beginning of the loop VII. This sequence might be important for the oligomerization of the peptide chain (see below). Interestingly, the tyrosine residue present in the β3 strand of plant sequences and thought to play an important role in the specificity of the reaction [14, 26], is not present in DIRLs (most of the time replaced by a cysteine).The alignment of DIRL sequences with plant DIR sequences of Additional file 1: Fig. S1 reveals a high conservation of glycine residues in predicted strands β2 to β7. Moreover, a glycine rich stretch of 13 residues (A/P-GGTG-Y/F—S/RG) is well conserved between the end of the β6 sheet and beginning of β7. Glycine are small residues providing flexibility to protein structures. Therefore, one can imagine DIRs as potentially adopting different conformations to adapt to substrate binding or product release.

The disulfide bridge stabilizing β1 and β8 β-sheets in several plant DIR (cysteines 40 and 186 in the AtDIR6 sequence [26], see Additional file 1: Fig. S1), is missing in bacterial DIRLs. However, most of DIRLs contain conserved cysteine residues in other locations, *e.g*., the very conserved cysteines found in β3 and β4 strands. If those cysteines are able to form a disulfide bridge and have a stabilizing or functional role in some DIRLs, it seems however not mandatory to the function of others having only one cysteine in their sequence (*S. alboflavus, S. albida, S. Silvensis* and *K. albida DSM 43870*).

We particularly looked at the nature of residues at locations corresponding to those proposed to form part of the pocket where radicals bind in Pinoresinol forming DIRs [32, 35], for which mutagenesis studies exist. Our sequence alignment reveals that in the β3 strand region aromatic residues are less present in DIRLs than in DIRs; in particular, a tyrosine pointed out as critical in plant DIRs seems to be replaced by a cysteine residue [14, 26]. As already pointed out most DIRLs hold two cysteine residues in the consecutive β3 and β4 strands. Cysteine residues play important role in proteins such as metal binding, electron donation, hydrolysis, and redox catalysis [33]. On the other hand, it is noteworthy that among the five DIRLs that do not have cysteines in the consecutive β3 and β4 strands, three (*S. alboflavus, S. formicae, S. silvensis*) form the group II of the phylogenetic tree of Fig. 3. Therefore, conserved residues of the β3 and β4 strands of DIRLs being different from those found in DIRs this leave open the possibility to have DIRLs involved in original radical coupling processes.

In the phylogenic tree of Fig. 3 shows a clear separation between the plant and bacterial kingdoms DIRLs are divided into several sub-families, exactly as for plant DIRs. Five sub-families are easily distinguishable. *Streptomycetaceae* are spread over three groups (I, II and V), *Deltaproteobacteria* cluster in group IV and *Gammaproteobacteria* in group III. Are these five families grouping DIRLs with the same substrate? Based on the consideration of amino acid conservation in particular in β3 and β4 strands this could be the case at least in group II.

Concerning the secondary structures, a β sheet organization similar to the one observed for plant DIRs is predicted for all DIRLs (Plant DIRs are mainly organized in a β -barrel made of up to 8 β strands [13, 25]. A similar structural organization is proposed for the DIRLs. From an analysis with the PredictProtein software, DIRLs would have a number of strands comprised between 4 and 8. A ninth strand, corresponding to the β1-bis strand proposed by Dabravolski [16] as well as by Meng et al*.* in 2020 [14] could be present in some DIRLs (Additional file 1: Fig. S1).

The 3D structure of few plant DIRs has been solved. The *At*DIR6 and DRR206 β strands form anti-sense sheets that arrange in a barrel shape [13, 32]. PsPTS1 or GePTS1 have a similar barrel structure with anti-sense leaflets but the N-terminal side leaves β strands on the outside of the barrel [13, 32, 35]. Plants DIRs assemble in a homotrimer structure where all monomers are joined in the same direction with the N-terminus and C-terminus on one side and loops on the other [35]. However, 3D structures predictions obtained with Alphafold2 suggest that majority of DIRLs have, as the plant DIRs, 8 β strands (see selection of models family in Fig. 4).

## Conclusion

Following the analysis of the genomic regions carrying DIRLs genes, it is important to emphasize that many of the genes surrounding DIRLs genes have not yet been characterized. This make the prediction on a biosynthetic pathway in which a DIRL could be involved difficult. The *Streptomycetaceae* family is the most represented among the 49 strains considered in this study. Combining predictions on secondary metabolism on AntiSMASH server (Table 1, column 4) and our analysis of loci organization and gene composition around DIRL coding genes, *S. formicae* KY appears as a good starting candidate. In this organism the gene encoding a DIRL cluster with terpenes biosynthesis genes (a terpene synthase and octaprenyl diphosphate synthase genes) and a cyt. P450 gene (Fig. 5). For others, close to the DIRL gene, we found potential operonic organizations. These operons are also surrounded by genes known in other bacteria to be involved in either the biosynthesis or the transportation of compounds or into the regulation of operonic gene expression (data not shown). *N. iriomotensis NBRC 105384* is surrounded by interesting markers: a cyt. P450 and an oxidoreductase (Additional file 2: Fig. S2). The two *Archangiaceae*, *S. aurantica* and *S. erecta* are a priori interesting for their known capabilities to synthesize antibiotics [36]. However, the genetic organization around the DIRL gene was not found remarkable enough to propose a clear link between the DIRL gene and antibiotic biosynthesis (Additional file 2: Fig. S2).

Beyond genomic organization and metabolic considerations another aspect has to be taken into account: the ability to grow the bacterium in a laboratory and to dispose of molecular tools to make mutant. Whereas investigation of laboratory growth conditions would be a precious help in identifying DIRLs function it is to note that only few bacteria among those listed in this study have been grown in a laboratory. Still, the CRISPR/Cas9 mutagenesis method has been developed recently for *S*. *formicae KY* that is grown in laboratory conditions [29]. This is particularly interesting since it is known that environmental growth conditions influence the secondary metabolism molecules expression. Slow progress in DIR studies lie in difficulties to identify substrate-product pairs for each DIR candidate. Testing multiple growth conditions help to increase DIR genes expression and facilitate identification into the cell. *S*. *formicae KY5* contains at least 45 secondary metabolism gene clusters such as formicamycin antibiotic operon [37]. Even if the DIRL gene is not localized in one of these clusters, this indicates the high potential of the bacteria in the synthesis of molecules of particular interest. The area of investigation regarding DIRL-mediated coupling reactions seems very large. In addition to the three known plant DIR mediated reactions, this might be a playground to look for the involvement of a DIRL in a radical coupling process. Identification of DIR like domains and their substrates in prokaryotes would bring novelties in the control of the coupling of radical moieties and would facilitate functional studies of the DIR proteins family. Analysis of DIR-mediated reactions in bacteria, together with the engineering of artificial DIRs with programmed specificity, will expand the scope of biosynthetic radical coupling reactions and their applications in organic and pharmaceutic synthesis.

## Supplementary Information

**Additional file 1: Figure S1**. Alignment of the 49 sequences of DIRLs from bacteria and five selected plant DIRs sequences in clustal0 on Uniprot server (Align tools). The plant DIRs are F. intermedia FiDIR1, P. sativum DRR206, G. echinata PTS1, A. Thaliana atDIR5 and A. Thaliana atDIR6 (AtDIR6, DRR206 and PTS1 have a resolved 3D structures and FiDIR1 and AtDIR5 are also well characterized).

**Additional file 2: Figure S2**. Schematic representation of a part of the genome of 25 bacteria, Genomes are annotated manually in order to obtain genomic information around the genes encoding a potential DIRL. With the NCBI number of the bacteria genomes in the Table 1, the fasta sequence of the genome is obtain and zoomed on the DPL region. Softberry server is used to predict the -10 and -30 regulation zone before the ATG of each predicted gene. For the other DPL, or the DPL genes is not well positioned on the contigue or not found (the sequenced genome for these bacteria is not complete or in not operonique structure.

**Additional file 3: Table S1**. Accessible Uniprot Accession number of DIRL from bacteria

**Additional file 4: Table S2**. Quantification of the 3D structural proximity of atDIR6 (LAL5) and each DIRL or well characterized DIR, with the use of aligned length residues, RMSD (Root Mean Square Deviation) calculation and Seq_ID = number of identical AA/ numbers of aligned AA. The plant DIRs are A. Thaliana atDIR5 and atDIR10, P. sativum DRR206, Glycin and G. echinata PTS1, and G. hirsutum GhDIR4 (DRR206 and PTS1 have a resolved 3D structure, the others are studied).
