# Supplementary material for: Investigation of dirigent like domains from bacterial genomes
Source: BMC Bioinformatics. 2022 Aug 2;23:313. doi: 10.1186/s12859-022-04832-6 (PMC9344732; doi:10.1186/s12859-022-04832-6)
Supplement: Supplementary file 1 — Additional file 1: Figure S1. Alignment of the 49 sequences of DIRLs from bacteria and five selected plant DIRs sequences in clustal0 on Uniprot server (Align tools). The plant DIRs are F. intermedia FiDIR1, P. sativum DRR206, G. echinata PTS1, A. Thaliana atDIR5 and A. Thaliana atDIR6 (AtDIR6, DRR206 and PTS1 have a resolved 3D structures and FiDIR1 and AtDIR5 are also well characterized). [file 12859_2022_4832_MOESM1_ESM.docx]

**Streptomyces_sp._Root1304** -----------------------------------------------------MSRSFR-----R-----FAVLGVC---TVAAAAFG-----------------A-VVPVAADSGDGFE 36

**Streptomyces_sp._PanSC19** -----------------------------------------------------MTRSSR-----R-----IGALCAC---TVAAAAFA-----------------AAPAPVSADDGDGFE 37

**Streptomyces_sp._62** -----------------------------------------------------MTRSFR-----R-----IGALCAS---TVAAVSFA-----------------AAAAPAHAAAGDGFE 37

Sphaerobacter_thermophilus ------------------------------------------------MRVALMRMTGRWVA--L-----VGAVAAVLAITSLLVVP----------------------RVAGQQGQTIV 43

Kutzneria_albida_DSM_43870 --------------------------------------------MA---TPAEL--SRRAAL--FAAPAVAAG-------GLVLAGSS---------------------PAAASANETIK 41

**Streptomyces_vietnamensis** -----------------------------------------------------MIRSLRTG---T-----LGVCTAA---AIAAVAFA-----------------AAPFSAFADDGEGFE 39

**Streptomyces_viridochromogenes** -----------------------------------------------------MTRTIR-----R-----IGTLGAC---TVAAVAFG-----------------A-VAPVQADNGDGFE 36

**Cystobacter_fuscus** -----------------------------------------------------MRK--AMF---L-----AVSVVSL-----TTPMLGCV------D--------------NAHAEESWT 32

Streptomyces_bottropensis -----------------------------------------------------MTM--KKF---RLAAVLVATVCVL-----AALGLSVA---------------QAEPQSSSRHGRATT 42

Nocardioides_iriomotensis -----------------------------------------------------MRS--KVV--------VAGTVATV-----VAGTVLT------------------V-SAAQGERAAPR 33

**Methylomicrobium_alcaliphilum** -----------------------------------------------------MFD--SLD---R-----LI-LLVY-----FLSMTACT------------------------HCMPKT 27

**Archangium_violaceum_Cb_vi76** ----------------------------------------------------------MLL---M-----A---VLT-----AAPMLSCV------N--------------NAPAEEAWT 26

Chloroflexi_bacterium_GWC2_73_18 -----------------------------------------------------MLR--RLF--------VVGAVVAL-----VAVAVSA------------------TALAAGDQAAGKT 34

Nocardia_mexicana -------------------------------------------------------MQHEKAI--FKRISI---GVAAA--SLLVGVT---------------SGCSEEQTSAASEPEVHE 43

**Gammaproteobacteria_bacterium_HGW** -----------------------------------------------------MSD--SL----G-----PI-LLVC-----FLSMTACT------------------------HSMPNT 26

**Cystobacter_fuscus_DSM_2262** -------------------------------------------------------------------------------------MLGCV------D--------------NVPAEESWT 15

**Archangium_gephyra** -----------------------------------------------------MRK--TIL---M-----A---VLT-----AAPMLSCA------N--------------NGPAEEAWT 29

**Cystobacter_ferrugineus** -------------------------------------------------------------------------------------MLGCV------D--------------NSQAEESWT 15

**Streptomyces_sp._SA15** -----------------------------------------------------MGFTRRPFP--FLAVAVLGGSAVL---ALI--LPASAEV-----ASQQTAIPAATLPADGSSEKSRT 55

**Stigmatella_erecta** --------------------------------------------MNKPKKTGKLQQ--AVQ---W-----AGGTLLL-----AAPGLGCA---------------------RAHADEPWT 40

**Streptomyces_alboflavus** -----------------------------------------------------MGNNSNRPL--FKRIAALSLATVAV--GVFAAGG---------------ANADA---SKKSRVEVIE 45

**Streptomyces_sp._NWU339** -----------------------------------------------------MGFAGRPLI--FLAIAVLGGSAGL---ALI--GSASADV-----IPLKTAETAATHPADGSSVKSRT 55

**Streptomyces_formicae** -----------------------------------------------------MENGKRRPL--LVKCAALSLVAAGV--GVLAVGS---------------AAADT---SGKDRAEVIE 45

**Streptomyces_sp._CB02400** -----------------------------------------------------MGFAGRPFI--FFAVAVLGCSAGL---TLI--GPASADA-----VPPETAPAAAPRPDDGSSAKSRA 55

**Streptomyces_caelestis** -----------------------------------------------------MGFAGRPFI--FLTVAVLGCSAGL---AVI--TSASADV-----APRKAAPTAAARPGDGSSVKSRA 55

Pseudonocardiales_bacterium -------------------------------------------------------M--RRS---RTIPLVLGGLAAA-----LAVGTSAVSLATSAD-------AATSAGSSNDRHWRTT 48

**Methylomicrobium_kenyense** -----------------------------------------------------MHG--HCF---R-----IA-YAGL-----LLCAVGCA------------------------RVVPQT 27

Streptomyces_griseoruber -----------------------------------------------------MTM--RKP---RLAAVLAATTCAV-----LVAGVSVA---------------QAGDRTAGHRGGPAV 42

Chloroflexi_bacterium -----------------------------------------------------MLSQRKLIA--LLALLML-------------ALSACTPV-----VPAATAGLTQTYAPGEAEAHVLE 47

**Thiogranum_longum** -----------------------------------------------------MHC--IFR---Y-----AA-TCAC-----ALLLGACA------------------------AEAPLQ 27

**Streptomyces_fungicidicus** -----------------------------------------------------MNIAGRPFI--FLAVAVLGCSAGL---ALI--GPASADV-----APRKAASTVAPHTDDGSPAKSRT 55

**Streptomyces_toyocaensis** -----------------------------------------------------MGFTGRPFI--FLAVAVLSCSAGT---ILI--DPASAGV-----VPSETAPAATPRPDDGSSARSRT 55

**Streptomyces_showdoensis** -----------------------------------------------------MTGSFR-----R-----MGALGAC---TAAVLALG-----------------T-AAPASADNGDGFE 36

Enterobacter_ludwigii -----------------------------------------------------MNR--KIV---A-----I---VAL-----LVAGITQA------T---------SSHNPETLRRNEIH 34

**Stigmatella_aurantiaca** --------------------------------------------MRKLKETGKMQQ--AVL---W-----AGCTLLL-----AAPGLGCS---------------------SAHADEPWT 40

Modestobacter_sp._DSM_44400 ------------------------------------------------------------------------------------------------------------------------

**Streptomyces_radiopugnans** -----------------------------------------------------MGFSARPLA--FLAAAVLGGSAGL---TLV--GPASAGA-----DPQKATVNAATRPADASPAKSRT 55

**Methylomicrobium_buryatense** -------------------------------------------------------------------------------------MTACT------------------------HCMPKT 11

**Vitiosangium_sp._GDMCC_1.1324** ----------------------------------------------------------------M-----AVFVVPF-----AVQGSGCS---------------------EAKAEESWT 25

**Archangium_sp._Cb_G35** ----------------------------------------------------------------M-----A---VVT-----AAPMLSCA------N--------------NAPAEEAWT 23

Cellulomonas_aerilata -------------------------------------------------------M--RRS---LTAALLAG-LGVL-----ALGGSSSAA-----D-------SADADRSSSGRAATLR 42

**Streptomyces_sp._NRRL_S-4** -----------------------------------------------------MGFAGRPFI--FLAVAVLGGSAGL---ATL--GSASADV-----VPQKAAATAEAHPADGSSEKSRT 55

**Streptomyces_silvensis** -----------------------------------------------------MENSSRRPL--LRKCAALSLVAAVV--GVLAVGS---------------AAADTSDTSAKDRTEVIE 48

**Streptomyces_sp._CNZ306** MTNFLDGAAGLLVRLRSHIRSAALPSVNRPTRRRPGKAWVALTLFIPPNTEATMRFARRHFT--FLAVAVFGGSAGL---TLN--GAASADA-----IPQKTAATAATHPGDGSSARGRT 108

**Streptomyces_sp._RV15** -----------------------------------------------------MKIGKRRIR--HAAL--ATCVATLTSLAIIAGGPSGDGQGDTKDETPPVAPRGDRPQKEVSPGEGRT 63

**Streptomyces_regalis** ------------------------------------------------------------------------------------------------------------------------

**Streptomyces_sp._13-12-16** -----------------------------------------------------MGFAGRPFM--FFAVAVLGCSAGI---ALI--GPASADV-----VPPETAPAAAPRPDDGTSAKSRA 55

Streptoalloteichus_hindustanus ----------------------------------------------------------------MNRTLAIAGAVALT--GVLAGAGLST-----------LGGPAAAETEAAGRAVTLD 43

**Hyalangium_minutum** ------------------------------------------------------------------------------------------------------------------------

**F.intermedia FiDIR1** ------------------------------------------------------MVSKTQIVALFLCFLT---S----------TSSATY----------------G--RKPRPRRPCKE 35

**P. sativum DRR206**  ------------------------------------------------------MGSKLPVL--FVFVML---F----------ALSSAI----------------P--NKRKPYKPCKN 33

**G. echinata PTS1**  -----------------------------------------------------MAKSTTFFISLTLPFLLLSVVTA--------TYYQSM----------------SPTVLGFQEEKFTH 43

**A.Thaliana atDIR5**  -------------------------------------------------------MVGQMKS--FLFLFVFLVLTK--------TVISAR----------------K--PSKSQPKPCKN 37

**A.Thaliana atDIR6**  ---------------------------------------------------MAFLVEKQLFKALFSFFLLVLLFSD--------TVLSF-----------------R--KTIDQKKPCKH 42

**IGS T M I**

**D S G FD L VGR G C VV C T L G L**

**Streptomyces_sp._Root1304** FTLYAK--EVPAP------------GGDQ------SGPPSKVGETFTFADDLYKTK--GGE--------------KVGRDGVICAVVRV--SGTSVEANCVGTF-----ELDGGPGGQLA 113

**Streptomyces_sp._PanSC19** FTLYAK--QIPPP------------ATSE------SNPPPKVGDTPVFADDLFRTK--GGD--------------KVGRDGVSCAVVRV--SGDQVDMNCVGTI-----VLNGGPGGQLT 114

**Streptomyces_sp._62** FTLYAK--QVPAA------------GASA------SMPAPKVGDVLTFADDLYKTK--GGD--------------KVGRDGVTCSVVRV--SGDQADMNCVGTI-----VLNGGPGGQLT 114

Sphaerobacter_thermophilus IDVAFD-QPNIHR-LAEAPA-----GAWP------ARGEVSSGGGKIFDGDLEATE-------------------QIGEFYFIGVGTSA-----PEYFETAANHLFEVARFELWGQGSID 126

Kutzneria_albida_DSM_43870 LVA--A--RSKNS----------------------LSATPAFGTPFAMQLELFDANAA-----------------AAGDGSAHGIVVNVTPDTPPKVI-VQMSL-----VLKLGFNGELH 112

**Streptomyces_vietnamensis** FTLYAK--QVPPP------------GTNE------SSPPPKVGDHPVFADDLYKTK--GGD--------------TVGRDGVACAVVRV--SGDQVDMNCVGTI-----LLNGGPGGQIA 116

**Streptomyces_viridochromogenes** LTLYAK--EVPAE------------GADE------SGPPPEVGDVFTVADDLYKTK--GGD--------------KVGRDGVTCAVVRV--SGTQADVNCVGTF-----VLNGGPGGQLT 113

**Cystobacter_fuscus** LTTIADARSGIASPVDM------------------APPGDSPGDMFVFDQPLLNEAK-----------------ENIGSNSGFCIRTLP-----GQFSECQWTL--------TMADGTIT 104

Streptomyces_bottropensis FTVIEH--ADTDTVVDL------------------GPRGDSIGDTLAFGNPIYDTAG-----------------NRAGDSQGSCVRTKV-----GTAWECSWTT--------TLNGGSIV 112

Nocardioides_iriomotensis ITVVEH--AVSDALVDL------------------APDGDSNGDLLTFANPVYDRTN---S-------------RKVGRDQGSCIRTVV-----GEAWQCSFTT--------WLGKGSLT 104

**Methylomicrobium_alcaliphilum** LITIADARTHQAKLVDI------------------GELGDTEGDILTFDQPLLDEQG-----------------SRIGNNSGMCIRTRV-----GHSFQCQWTL--------TMTNGSIQ 99

**Archangium_violaceum_Cb_vi76** LNTIADARSGIATPVDL------------------GPPGDSPGDMFVFDQPLLNEAK-----------------ETIGSNSGYCIRTLP-----GQFSECQWTL--------TMAEGTIT 98

Chloroflexi_bacterium_GWC2_73_18 LRVVEH--AVSDSVVDL------------------APSGDSLGDVLAFGNPIFDAAD---D-------------HEIGRDQGYCVRTNV-----GEAWECNWTV--------ILARGSIT 105

Nocardia_mexicana LQV----TTDQYSALDL------------------GSSGTSVGDMDVFSGSAMKDGS------------------KVGNAGGSCQATHV--DGEKVTTQCLITM--------ELEAGALA 113

**Gammaproteobacteria_bacterium_HGW** LITIADARTHQAKLVDI------------------GELGDTEGDILTFDQPLLDEQG-----------------SQIGNNSGICIRTRV-----GHSFQCQWTL--------TMKNGSIQ 98

**Cystobacter_fuscus_DSM_2262** LTTIADARSGIASPVDL------------------APPGDSPGDMFVFDQPLLNAAK-----------------ENIGSNSGFCIRTLP-----GQFSECQWTL--------TMADGTIT 87

**Archangium_gephyra** LNTIADARSGIATPVDV------------------DPPGDSPGDMFVFDQPLLNEAK-----------------ETIGSNSGYCVRTLP-----GQFSECQWTL--------TMADGTIT 101

**Cystobacter_ferrugineus** MTTIADARSGIASSVDL------------------GTPGDSPGDMFVFDQPLLNAAK-----------------ENIGSNSGFCIRTLP-----GQFSECQWTL--------TMADGTIT 87

**Streptomyces_sp._SA15** IRV--VAGLQVGEELDL------------------GAPGRSVGDQFIFSGNLSSTRGSEEE-------------RVVGRFGGFCVITDLE----RNAGQCSSTA--------VLPEGQIT 130

**Stigmatella_erecta** FTTIADARSGIATPVDL------------------GAPGDSPGDMFVFDQPLLNEAK-----------------QNIGSNSGFCVRTLP-----GQFNECQWTL--------TLADGTIT 112

**Streptomyces_alboflavus** LQL----KDLEYESFDL------------------GKPGPSLGDMTVYCATAVENGR------------------NVGRGAGTSQVVSA--RGGKVTSQAVITI--------ELKRGSLT 115

**Streptomyces_sp._NWU339** IRV--EARLQAGEELDL------------------GATGRSVGDQFVFSGNLVSTEGPE-E-------------RFVGRFGGFCVITDLE----CNAGQCSSTA--------VLPEGQIT 129

**Streptomyces_formicae** LQI----KDLEYKAIDV------------------GPTGPGLGDMSVFCGTAVENGR------------------TVGRGAGTSQVVSV--DGERHTSQAVITI--------ELERGSLT 115

**Streptomyces_sp._CB02400** IRV--EARLHVGEELDL------------------GAAGRSVGDQFVFSGNLTSTEGRE-E-------------RVVGRIGGFCVITDLE----RNAGQCASTA--------VLPEGQIT 129

**Streptomyces_caelestis** IRV--EARLRVGEELDL------------------GATGRSIGDQFVFSGDLSSAEGAG-E-------------RLVGRIGGFCVITDLE----RNAGPCTSTA--------VLPEGQIT 129

Pseudonocardiales_bacterium LKFVEH--ETGASQADL------------------PPAGPSVGDNFFGTNPLFNESD---T-------------RQVGRQVGFCTQASKT---TPAVLYCDLTY--------QLANGMIT 121

**Methylomicrobium_kenyense** LVTIADARADKARVTDL------------------GDPGDSPGDILTFDQPLLDTRQ-----------------KPIGSNSGFCVRTRV-----GHSFQCQWTL--------TLQDGSIQ 99

Streptomyces_griseoruber FTVIEH--ADTDTVVDL------------------GPRGDSIGDTLAFGNPVYDTAG-----------------HQVGGSQGSCVRTKV-----GTAWECSWTT--------TLNGGSIV 112

Chloroflexi_bacterium FDVAEDMNRFIFD-QDV----------VY------DDGMPADGSSFITRGYLYEPGTLNGSNGVNPDGSPEFPDKVIGEW--ICQGYMI--NDAGHATGGVWVFSTQFFQLGTEPGAQTV 146

**Thiogranum_longum** LVTLADARTDKAQVIDT------------------GEPGDSVGDILVFDQPLLDEHK-----------------QPVGNNSGACIRTRV-----AHSFQCQWTL--------SLDGGTIQ 99

**Streptomyces_fungicidicus** IRV--EAQLQVGEEIDL------------------GATGRSVGDQFVFSGNLMSTEGTE-G-------------HSVGRIGGFCVIDDLE----RNAGQCVSTA--------VLAGGQIT 129

**Streptomyces_toyocaensis** IHV--QARLHVGEELDL------------------GAAGRSVGDQFVFSGDLTSSEGAE-E-------------RVVGRISGFCVITDLE----RNAGQCASTA--------VLPEGQIT 129

**Streptomyces_showdoensis** FTLYAK--EVPGP------------TASE------SAPAPKLGESFAFADDLYKTKDAGGD--------------KVGRDGGICAVVRT---GDPMDLQCVGTV-----VLNGGPGGQLA 114

Enterobacter_ludwigii IITIADGTRGEKRTVNT------------------GQNGPSPGDLFVFDQPLMDQHR-----------------RDIGTNSGYCVTTKI-----GVHSQCQWTL--------KTYGSTVV 106

**Stigmatella_aurantiaca** FTTIADARSGIANSVDL------------------GAPGDSPGDMFVFDQPLLNEAK-----------------QNIGSNSGFCVRTLP-----GQFNECQWTL--------TLADGTIT 112

Modestobacter_sp._DSM_44400 -------------------------MDGE------EDESAPVGSRLFISEALYETDDGT-T-----------RGDEVGRTHIECTAQVY-----DFTFACDIAFVF---DSGSQLHGSVV 69

**Streptomyces_radiopugnans** IRV--EARLQVGEELDL------------------GTTGRSVGDQFVFSGNLVSTEGPE-E-------------RPVGRFGGFCVITDLE----RNAGQCSSTA--------VLPEGQIT 129

**Methylomicrobium_buryatense** LITIADARTHQAKLLDI------------------GELGDTEGDILTFDQPLLDEQG-----------------SRIGNNSGMCIRTRV-----GHSFQCQWTL--------TMKNGSIQ 83

**Vitiosangium_sp._GDMCC_1.1324** LTTIADARSGIASPVDL------------------GAPGDSPGDMFVFDQPLLDEAK-----------------QTIGSNSGYCVRTLP-----GQFSECQWTL--------TMADGTIT 97

**Archangium_sp._Cb_G35** LNTIADARSGIATPVDL------------------GPPGDSPGDMFVFDQPLLNEAK-----------------ETIGSNSGYCIRTLP-----GQFSECQWTL--------TMADGTIT 95

Cellulomonas_aerilata YD-VEF--TSGFFLLDFGPHGVREVASFQ------DPFSPSRGDQVVFEDTLLRRGE------------------PVASGGGTCTVTAVVPSDTPLRLSCVATY--------ELPGGQVA 127

**Streptomyces_sp._NRRL_S-4** IRV--EARLQAGEELDL------------------GATGRSVGDQFVFSGNLASTGGPA-E-------------RSVGRFGGFCVITDLE----RNAGQCSSTA--------VLPGGQIT 129

**Streptomyces_silvensis** LQM----RDLEQKALDL------------------GPKGPSLGDMSIFCATVVENGR------------------TVGRGAGTAQVVYL--DGGRRTSQAVITI--------ELERGSLT 118

**Streptomyces_sp._CNZ306** ISL--EAQLQVGEELDL------------------GATGRTVGDQFIFSGNLVSTEGQG-E-------------RPVGRFGGFCVITDLE----RNAGQCSSTA--------VLPEGQIT 182

**Streptomyces_sp._RV15** IQV--EANVQVAEELDL------------------GAPGRSVGDQFVFSGNLSSAEDP--D-------------RVIGRFSEFCVITDLE----RNAGQCMLTA--------VLPGGQIA 136

**Streptomyces_regalis** --M--EANVQTGEELDL------------------GAPGRSIGDQFVFRGSLSPADEP--D-------------RVIGRFSEFCVISDLE----RNAGPCMLTA--------VLEGGQIA 71

**Streptomyces_sp._13-12-16** IHV--EARLRVGEELDL------------------GAAGRSVGDQFVFSGDLTSAEGGG-E-------------SVIGRIGGFCVITDLE----RNAGQCASTA--------VLPEGQIT 129

Streptoalloteichus_hindustanus LHV----ANDQFAAHDI------------------GQPGKSLGDMTVYSDKMVRDGR------------------EVGEDGGTCVVTHV--DGPRSISNCVLTI--------RLAEGQLT 113

**Hyalangium_minutum** --------------------------------------------MFVFDQPLLNEAG-----------------QNIGSNSGFCVRTLP-----GQFSECQWTL--------TMSDGSIT 46

**-A -L -K +G|-A +LLF|-FYL**

**F. intermedia FiDIR1** LVFYFH--DVLFKGNNYHNATSAIVGSPQWGNKTAMAVPFNYGDLVVFDDPITLDNNLH--------------SPPVGRAQGMYFYDQKN------TYN-AWLGFSFLFNSTKYVGT-LN 131

**P. sativum DRR206** LVLYFH--DILYNGKNAANATSAIVAAPEGVSLTKLAPQSHFGNIIVFDDPITLSHSLS--------------SKQVGRAQGFYIYDTKN------TYT-SWLSFTFVLNSTHHQGT-IT 129

**G. echinata PTS1** LHFYFH--DVVTGPKP----SMVIVAEPNGKAK----NSLPFGTVVAMDDPLTVGPESD--------------SKLVGKAQGIYTSISQE------EMG-LMMVMTMAFSDGEFNGSTLS 132

**A.Thaliana atDIR5** FVLYYH--DIMFGVDDVQNATSAAVTNPPGLG------NFKFGKLVIFDDPMTIDKNFQ--------------SEPVARAQGFYFYDMKN------DYN-AWFAYTLVFNSTQHKGT-LN 127

**A.Thaliana atDIR6**  FSFYFH--DILYDGDNVANATSAAIVSPPGLG------NFKFGKFVIFDGPITMDKNYL--------------SKPVARAQGFYFYDMKM------DFN-SWFSYTLVFNSTEHKGT-LN 132

**Pocket A and B from Active Site**


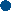

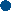


**β1** loop I **β1*bis* loop II β2 loop III (Ω) β3 loop IV β4 loop V**

**A Y S**

**P GGTG F RG**

**Streptomyces_sp._Root1304** GQTLATFDTSAGAPAAFDVAVTGGTGDFKNARGYIRSTPDG---DYE----RMEFHINVR------------------------- 166

**Streptomyces_sp._PanSC19** AQALTTFDTSNDSPSAFDLAVTGGTGDFKDARGYIRSTPDG---DYD----RMEFHINTR------------------------- 167

**Streptomyces_sp._62** AQALATVDITNEAPAALDIAVTGGTGDFKQARGYIRSTPDG---DYD----RMEFHITTR------------------------- 167

Sphaerobacter_thermophilus VMGTVTF---RG---TSHLSITGGTGAFAMARGECTEEMVA---SR---RGRFTCYIER-------------------------- 173

Kutzneria_albida_DSM_43870 LSTEHPL--VLPSPVDNPLAIVGGCGKYATARGEGTISYPS---PD---RINLTLNVRTD------------------------- 164

**Streptomyces_vietnamensis** AQALTTFDTSNDTPSAFDLAVTGGTGDFKSARGYIRVTPDG---DYD----RMDFHITKR------------------------- 169

**Streptomyces_viridochromogenes** AQALVTFDTSAEAPAAYDVAITGGTGDFKDARGYVRTTPDG---DYE----RMEFHINVR------------------------- 166

**Cystobacter_fuscus** VAGREAE---SG---TSLIPIVGGTGAYVGASGVLTTTPNG---DR---TFTQVLTFLKPKQ----------------------- 154

Streptomyces_bottropensis AQGPFYD---TA---DSTLAITGGTGKWRTARGQMQLHARD---AKG-SAYDFTFAVKR-------------------------- 161

Nocardioides_iriomotensis VEGPFYD---AH---PSVMAITGGTGAYARARGVMHLAARN---SQG-TAFTFRFVIR--------------------------- 152

**Methylomicrobium_alcaliphilum** VAGREFD---KG---TSVIAIVGGTGRYSGISGQMESVNNG---DG---TFTQILHYWVQ------------------------- 147

**Archangium_violaceum_Cb_vi76** VAGREAE---TG---TSLIPIIGGTGTFEGASGVLSTTPNG---DR---TFTQVLTLLKPKK----------------------- 148

Chloroflexi_bacterium_GWC2_73_18 VEGPFYD---DL--RDSPLAITGGTGAYLNARGQMTLHARN---AGG-TAFDFIYHIVG-------------------------- 155

Nocardia_mexicana VQALWIK---GT--SPLDMAITGGTGAYRNARGVVRFWDIA---TP---NERLRTEIFY-------------------------- 161

**Gammaproteobacteria_bacterium_HGW** LAGREFD---TG---TSVIAIVGGTGQYSAISGQMESVNNG---DG---TFTQILHYWVQ------------------------- 146

**Cystobacter_fuscus_DSM_2262** VAGREAE---TG---PSSIPIVGGTGAYVGASGVLLTTPNG---DR---TFTQVLTFLKPKQ----------------------- 137

**Archangium_gephyra** VAGREAE---TG---TSLIPIIGGTGTYEGASGVLTTTPNG---DR---TFTQVLTLLKPKK----------------------- 151

**Cystobacter_ferrugineus** VAGREAE---SG---PSSIPIIGGTGAYEGASGVLLTTPNG---DR---TFTQVLTFLKPKQ----------------------- 137

**Streptomyces_sp._SA15** VQGEQIG---IPVPSPVSNAITGGTGEFRKARGQVTQRVLT---PA---TWQLTFEVLDVQPSRSRDGLNRSQQPTPTPPSFPGK 206

**Stigmatella_erecta** VAGRESD---TG---TSMIPVAGGTGNYLGVKGVLATTPNG---DR---TFTQVLSLIKAAP----------------------- 162

**Streptomyces_alboflavus** MQSLRPE---EA--SSVDMAITGGTGAFKDARGSVRYWDIN---TP---QERLRAEILH-------------------------- 163

**Streptomyces_sp._NWU339** VQGEQAG---IPVPSPVVDAITGGTGEFRKARGQVTQRVLT---PA---IWQLTFEVFDVQPHRTGDGRSPTKVPMRMPPSFPGK 205

**Streptomyces_formicae** MQSLQAS---DA--RSLDMAITGGTGAFKDARGTARYWDIA---TP---QERMRAEILH-------------------------- 163

**Streptomyces_sp._CB02400** VQGEQAG---IPVPVPVVNAITGGTGEFRRAHGQVTQRVLT---PA---TWQLTFELSDVRPHRTEDGRGPAEAPTPVRPSFPGK 205

**Streptomyces_caelestis** IQGEQAG---IPVPAPVVNAITGGTGEFRKARGQVTQRVLT---PA---TWQLTFEVADVPPHRAEDGRSPSAAPMPVSPSFLGK 205

Pseudonocardiales_bacterium VHGIYDV---AA--GKVVSAVTGGTGAYVNARGEAISVFGP---VKSTHSITLVF------------------------------ 168

**Methylomicrobium_kenyense** VAGREFD---EG---SSDIAIVGGTGEYACIRGQVESVNNG---DG---TFTQTLHYWIDCR----------------------- 149

Streptomyces_griseoruber VQGPFHD---AA---DSTLAITGGTGKWRTARGQMHLHARD---AKG-SAYDFTFAVER-------------------------- 161

Chloroflexi_bacterium VTQGYEL---ADTDVSIARAITGGTGEFKLARGESTQTLLG---LNATEGVNLRVQIAVEK------------------------ 201

**Thiogranum_longum** VAGREFD---TG---ISTMSIVGGTGIYAGIRGDMESINNN---DG---TFTQTLRYRLMP------------------------ 148

**Streptomyces_fungicidicus** IQGEQAG---IPAPSPVVNAITGGTGEFRKARGQVAQRVLT---PA---TWRLTFEVSDVQSHQAGDARDPFEVPMPMPPSFPGK 205

**Streptomyces_toyocaensis** VQGEQTG---IPVPAPVVNAITGGTGEFGTAHGQLRQRVLT---PA---TWQLTYQLSDVRPHRPEDGRSPAKEPTPAPPSRPGK 205

**Streptomyces_showdoensis** LQTLAAVDPDDEAPPALDIAITGGTGDFENARGWVRSTPDG---DWS----RMDFHIATR------------------------- 167

Enterobacter_ludwigii VAGQEAE---SG---ISILAVIGTTGKFSGFMGEMTSEPNG---DG---TFTQKLTLFRP------------------------- 154

**Stigmatella_aurantiaca** VAGRESD---TG---TSMIPVVGGTGNYLGVKGMLATTPNG---DR---TFTQVLSLIKATP----------------------- 162

Modestobacter_sp._DSM_44400 VDFSTQS---ETEALQFDIAVTGGTGDYSRAKGVVNLLDIS---EDPEAE-TETLYE----AHRG-------------------- 123

**Streptomyces_radiopugnans** VQGEQTG---IPAPGPVTNAITGGTGEFRKARGQVTQRVLT---PA---TWRLTFEISDVRPHRAGGGHSLPEVSAPKPPSLTGK 205

**Methylomicrobium_buryatense** VAGREFD---TG---TSAIAIVGGTGQYSGISGQMESVNNG---DG---TFTQILHYWVQ------------------------- 131

**Vitiosangium_sp._GDMCC_1.1324** VAGREAE---NG---TSYIPVIGGTGTYLGVSGVLATTPNG---DR---TFTQVLTLLKAK------------------------ 146

**Archangium_sp._Cb_G35** VAGREAE---TG---TSLIPIIGGTGTFEGASGVLSTTPNG---DR---TFTQVLTLLKPKK----------------------- 145

Cellulomonas_aerilata VQGRTTN---AP---EKTLAVVGGTGRYAGAAGEFTLTEFG---DG---TGSAVFRLRR-------------------------- 174

**Streptomyces_sp._NRRL_S-4** VQGEQAG---IPVPKTVVNAITGGTGEFRKARGQVTQRVLT---PA---TWQLTFEVSDMQPHRARDGRIPSEAP--TPPSFPGK 203

**Streptomyces_silvensis** MQALQPS---DA--RSLDMAITGGTGAFRDARGTAHYWDIA---TP---KERMRAEILH-------------------------- 166

**Streptomyces_sp._CNZ306** VQGEQAG---IPVPSPVVNAITGGTGEFRKARGQVTQRVLT---PS---TWKLTFEVLDVQHRRTGDGPTPSEVPMPVPPTSPGK 258

**Streptomyces_sp._RV15** VQGEQEG---IPTPTSVTNAITGGTGEFRDAQGQMTLKVLT---AA---TWAITFQVTDH------------------------- 187

**Streptomyces_regalis** VQGEQDG---IPTPTSATNAITGGTGKFRKAQGEMTLEVLS---AA---TWVITFQLADR------------------------- 122

**Streptomyces_sp._13-12-16** VQGEQTG---IPVPVPVVNAITGGTGEFRRAHGQVTQRVLT---PA---TWQLTFELSGVRPHRTEDGRGPAEAPTPVHPSSPGK 205

Streptoalloteichus_hindustanus AQGLWDD---GT--SPMQMAITGGTGAYRNARGYLQAWDIH---TP---KERYRIQVSL-------------------------- 161

**Hyalangium_minutum** VAGREAE---TG---PSQIPVIGGTGAYLGVSGVLVTTPNG---DR---TFTQVLTLIRPNS----------------------- 96

**-I|+F +P -M -L -F -M**

**F. intermedia FiDIR1**  FAG-ADP----LLNKTRDISVIGGTGDFFMARGVATLM---TDAFEGDVYFRLRVDINLYECW---------------------- 186

**P. sativum DRR206**  FAG-ADP----IVAKTRDISVTGGTGDFFMHRGIATIT---TDAFEGEAYFRLGVYIKFFECW---------------------- 184

**G. echinata PTS1** ILARNMI----MSEPVREMAIVGGTGAFRFARGYAQAKFYSVDFTKGDAIVEYDIFVFHY------------------------- 188

**A.Thaliana atDIR5**  IMG-ADL----MMVQSRDLSVVGGTGDFFMSRGIVTFE---TDTFEGAKYFRVKMDIKLYECY---------------------- 182

**A.Thaliana atDIR6** IMG-ADL----MMEPTRDLSVVGGTGDFFMARGIATFV---TDLFQGAKYFRVKMDIKLYECY---------------------- 187

**β5 loop VI β6 loop VII β7 loop VIII β8**

Legend: X -> Amino acids involved in the formation of (+)-pinoresinol in lignans forming DPs

X -> Amino acids involved in the formation of (-)-pinoresinol in lignans forming DPs

X -> Amino acids particularly preserved between plant DPs and DPLs (often Glycines)

X/X -> Notorious conserved amino acids

C -> Cysteines

C -> Cysteines predicted in the formation of disulfide bonds

XXXX -> Amino acids constituting the predicted signal peptide

: loop between β-sheet on AtDIR6 protein sequence

:beta strand localization on AtDIR6 protein sequence

**Groupe I, Groupe II, Groupe III, Groupe IV and Groupe V** on the phylogenetic tree. Selected **DPs from plants**

*Figure S1: Alignment of the 49 sequences of DIRLs from bacteria and five selected plant DIRs sequences in clustal0 on Uniprot server (Align tools). The plant DIRs are* F. intermedia FiDIR1, P. sativum DRR206, G. echinata PTS1, A. Thaliana atDIR5 and A. Thaliana atDIR6 *(*AtDIR6, DRR206 *and* PTS1 *have a resolved 3D structures and* FiDIR1 *and* AtDIR5 *are also well characterized).*
