# Supplementary material for: Investigation of dirigent like domains from bacterial genomes
Source: BMC Bioinformatics. 2022 Aug 2;23:313. doi: 10.1186/s12859-022-04832-6 (PMC9344732; doi:10.1186/s12859-022-04832-6)
Supplement: Supplementary file 2 — Additional file 2: Figure S2. Schematic representation of a part of the genome of 25 bacteria, Genomes are annotated manually in order to obtain genomic information around the genes encoding a potential DIRL. With the NCBI number of the bacteria genomes in the Table 1, the fasta sequence of the genome is obtain and zoomed on the DPL region. Softberry server is used to predict the -10 and -30 regulation zone before the ATG of each predicted gene. For the other DPL, or the DPL genes is not well positioned on the contigue or not found (the sequenced genome for these bacteria is not complete or in not operonique structure. [file 12859_2022_4832_MOESM2_ESM.pptx]

## Slide 1
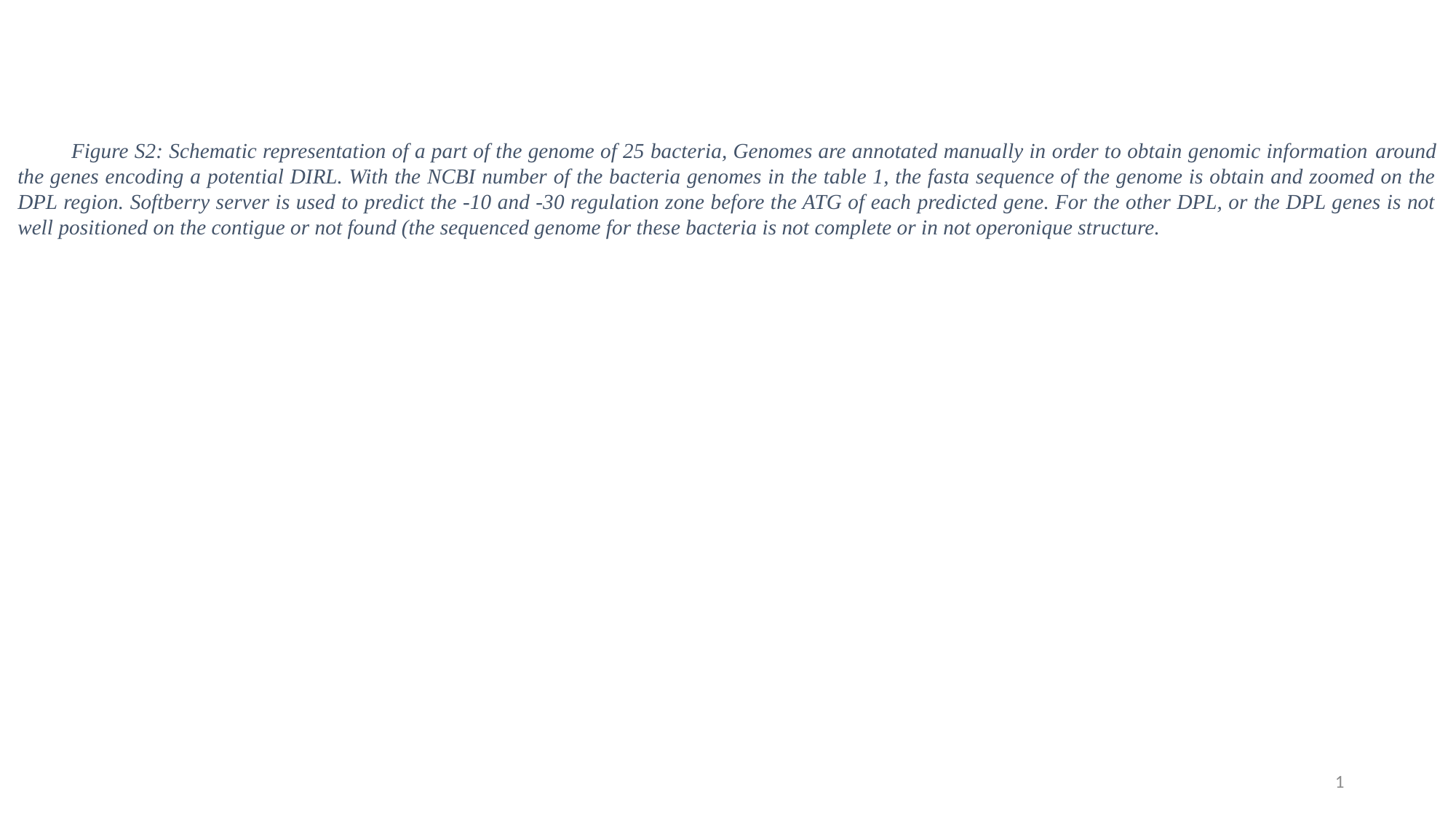

Figure S2: Schematic representation of a part of the genome of 25 bacteria, Genomes are annotated manually in order to obtain genomic information around the genes encoding a potential DIRL. With the NCBI number of the bacteria genomes in the table 1, the fasta sequence of the genome is obtain and zoomed on the DPL region. Softberry server is used to predict the -10 and -30 regulation zone before the ATG of each predicted gene. For the other DPL, or the DPL genes is not well positioned on the contigue or not found (the sequenced genome for these bacteria is not complete or in not operonique structure.
1

## Slide 2
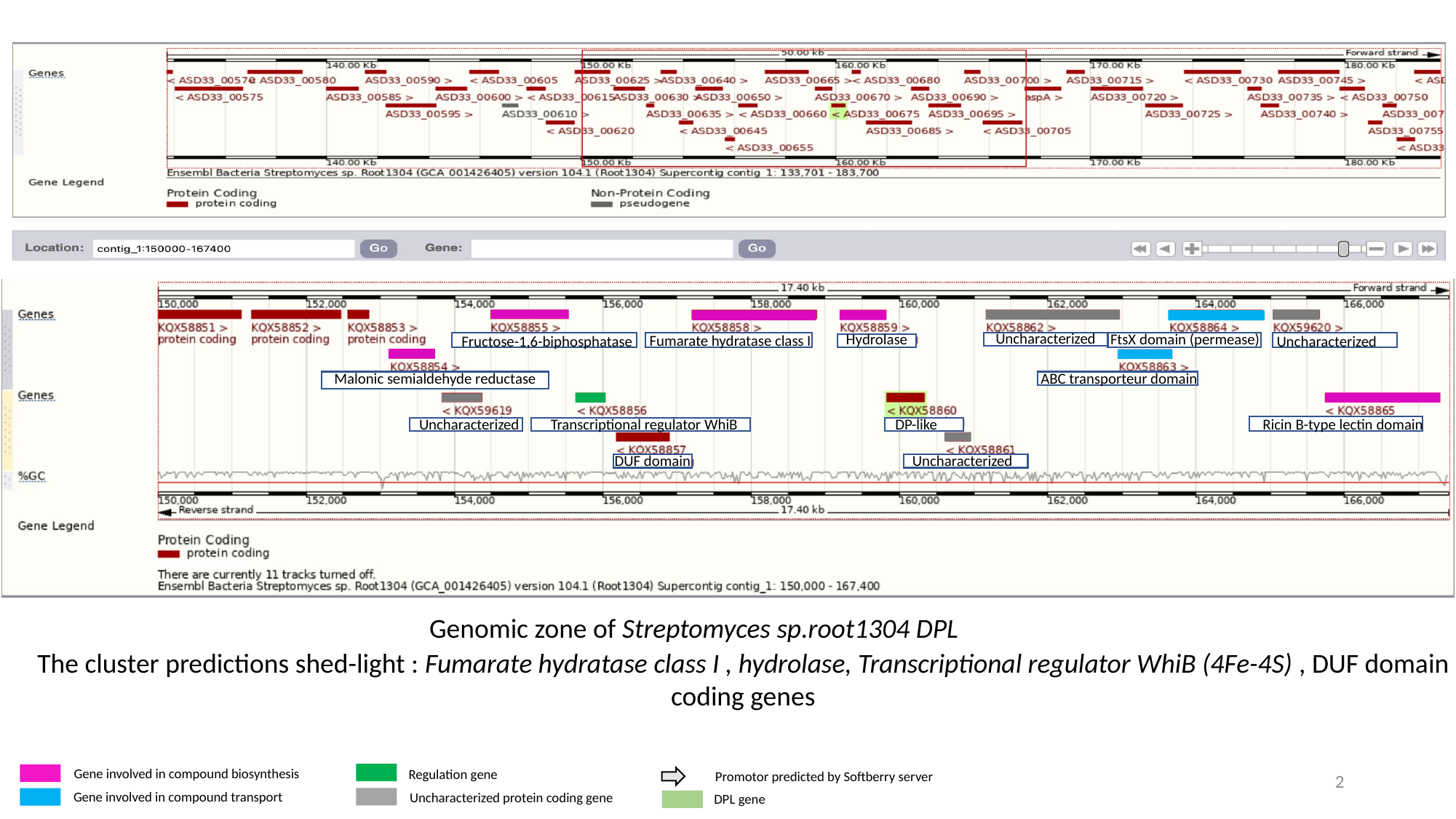

Uncharacterized
Hydrolase
FtsX domain (permease)
Fumarate hydratase class I
Fructose-1,6-biphosphatase
Uncharacterized
ABC transporteur domain
Malonic semialdehyde reductase
Transcriptional regulator WhiB
Uncharacterized
Ricin B-type lectin domain
DP-like
Uncharacterized
DUF domain
Genomic zone of Streptomyces sp.root1304 DPL
The cluster predictions shed-light : Fumarate hydratase class I , hydrolase, Transcriptional regulator WhiB (4Fe-4S) , DUF domain coding genes
2
Gene involved in compound biosynthesis
Regulation gene
Promotor predicted by Softberry server
Gene involved in compound transport
Uncharacterized protein coding gene
DPL gene

## Slide 3
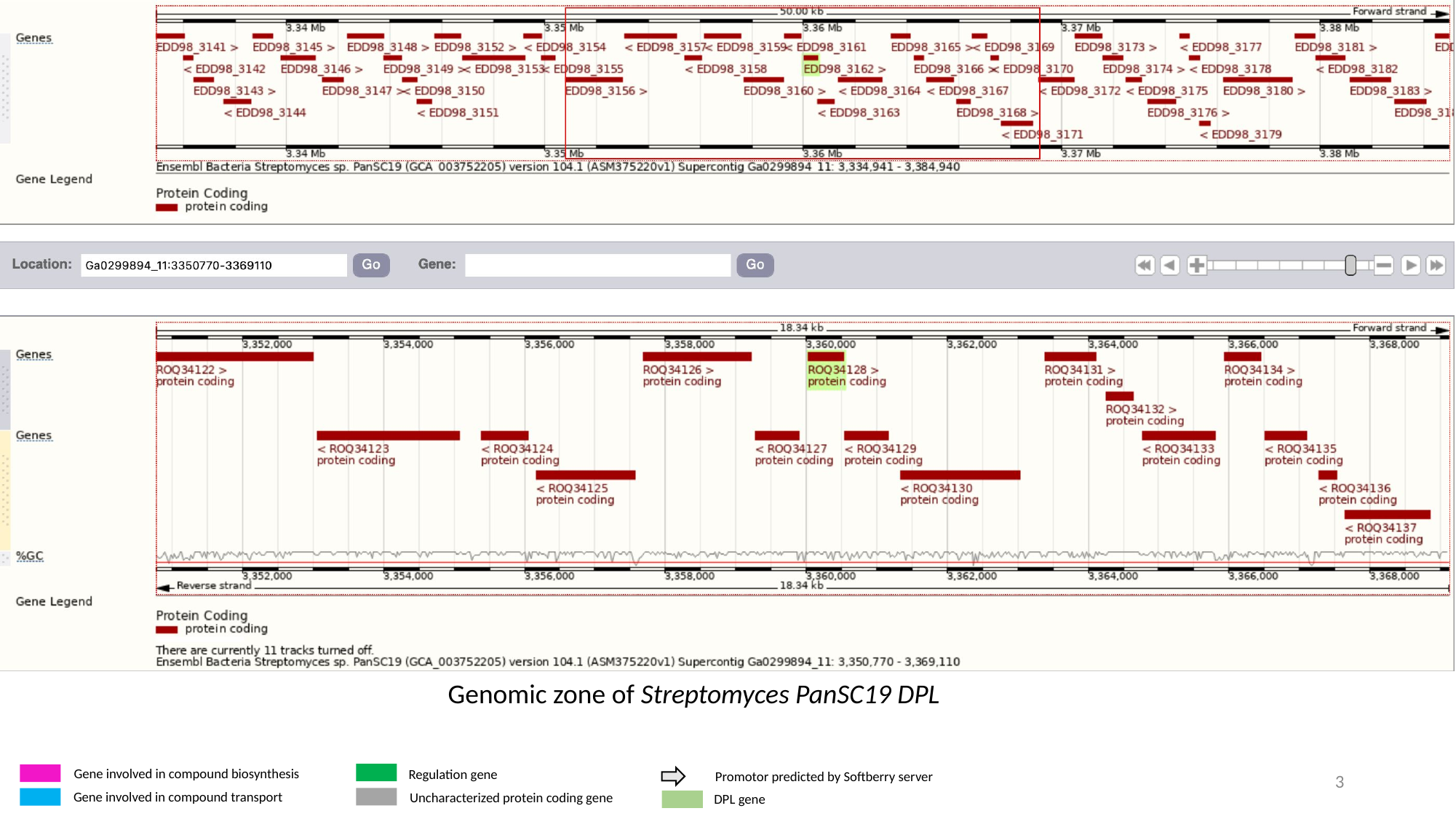

Genomic zone of Streptomyces PanSC19 DPL
3
Gene involved in compound biosynthesis
Regulation gene
Promotor predicted by Softberry server
Gene involved in compound transport
Uncharacterized protein coding gene
DPL gene

## Slide 4
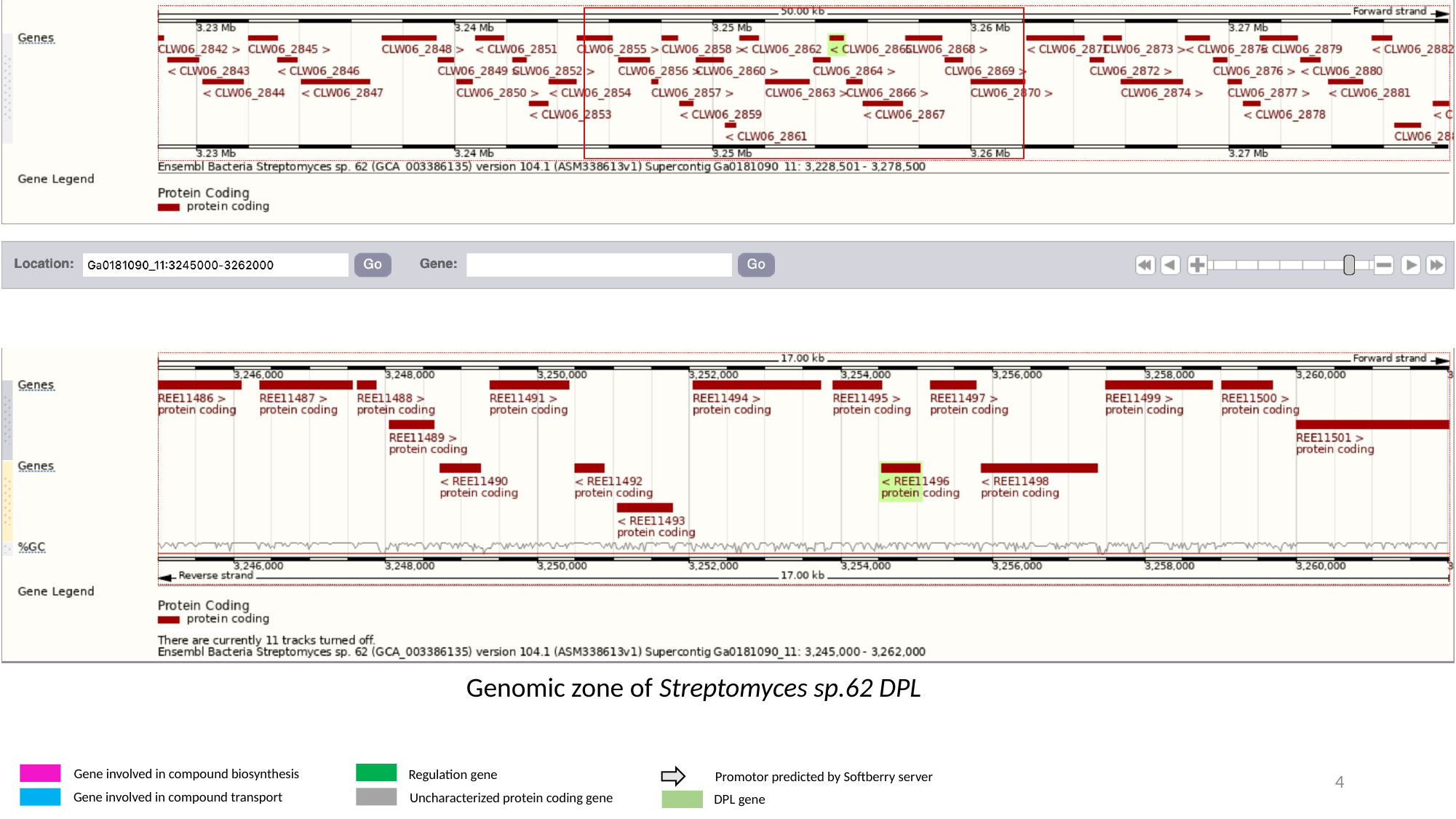

Genomic zone of Streptomyces sp.62 DPL
4
Gene involved in compound biosynthesis
Regulation gene
Promotor predicted by Softberry server
Gene involved in compound transport
Uncharacterized protein coding gene
DPL gene

## Slide 5
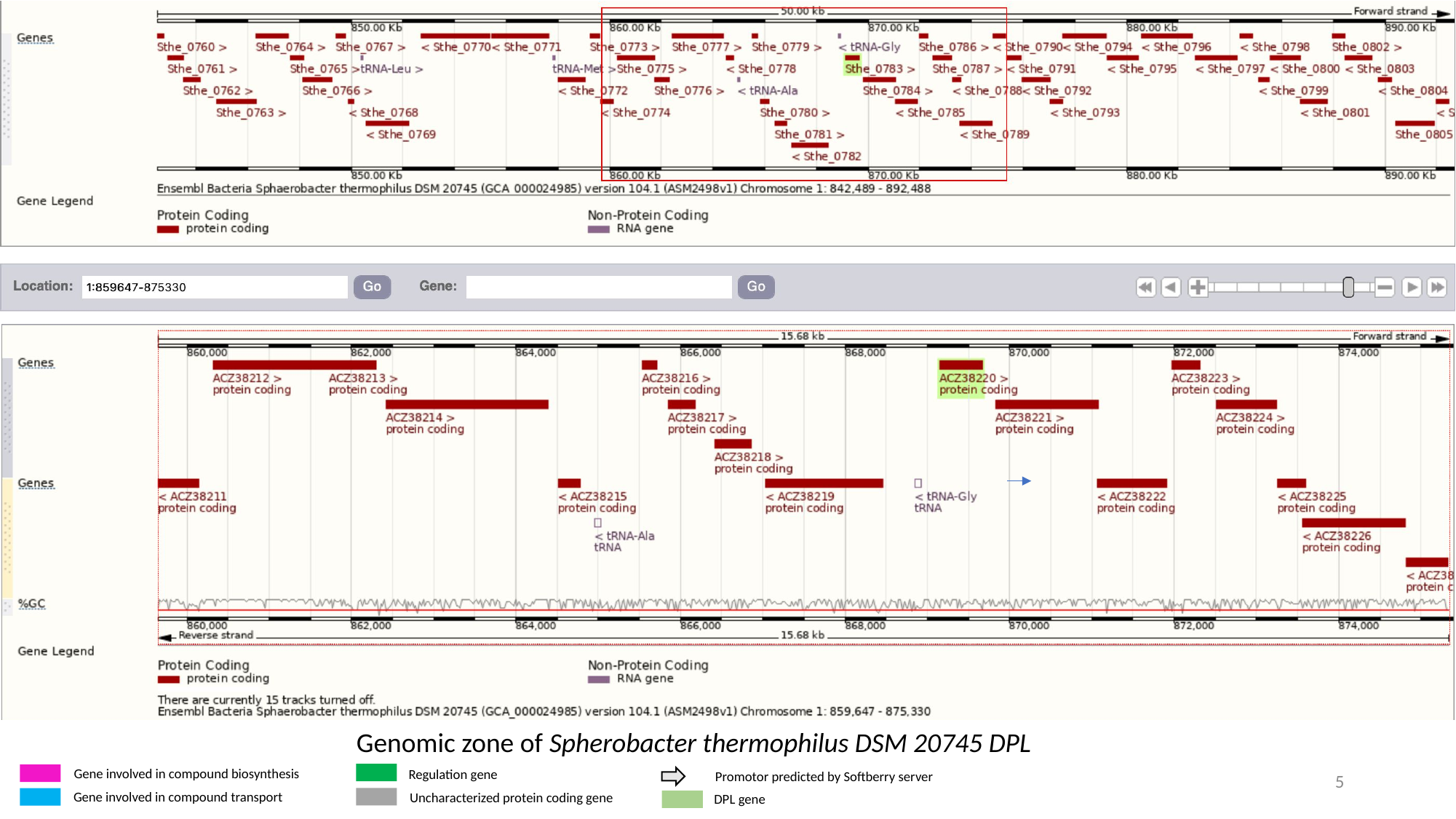

Genomic zone of Spherobacter thermophilus DSM 20745 DPL
5
Gene involved in compound biosynthesis
Regulation gene
Promotor predicted by Softberry server
Gene involved in compound transport
Uncharacterized protein coding gene
DPL gene

## Slide 6
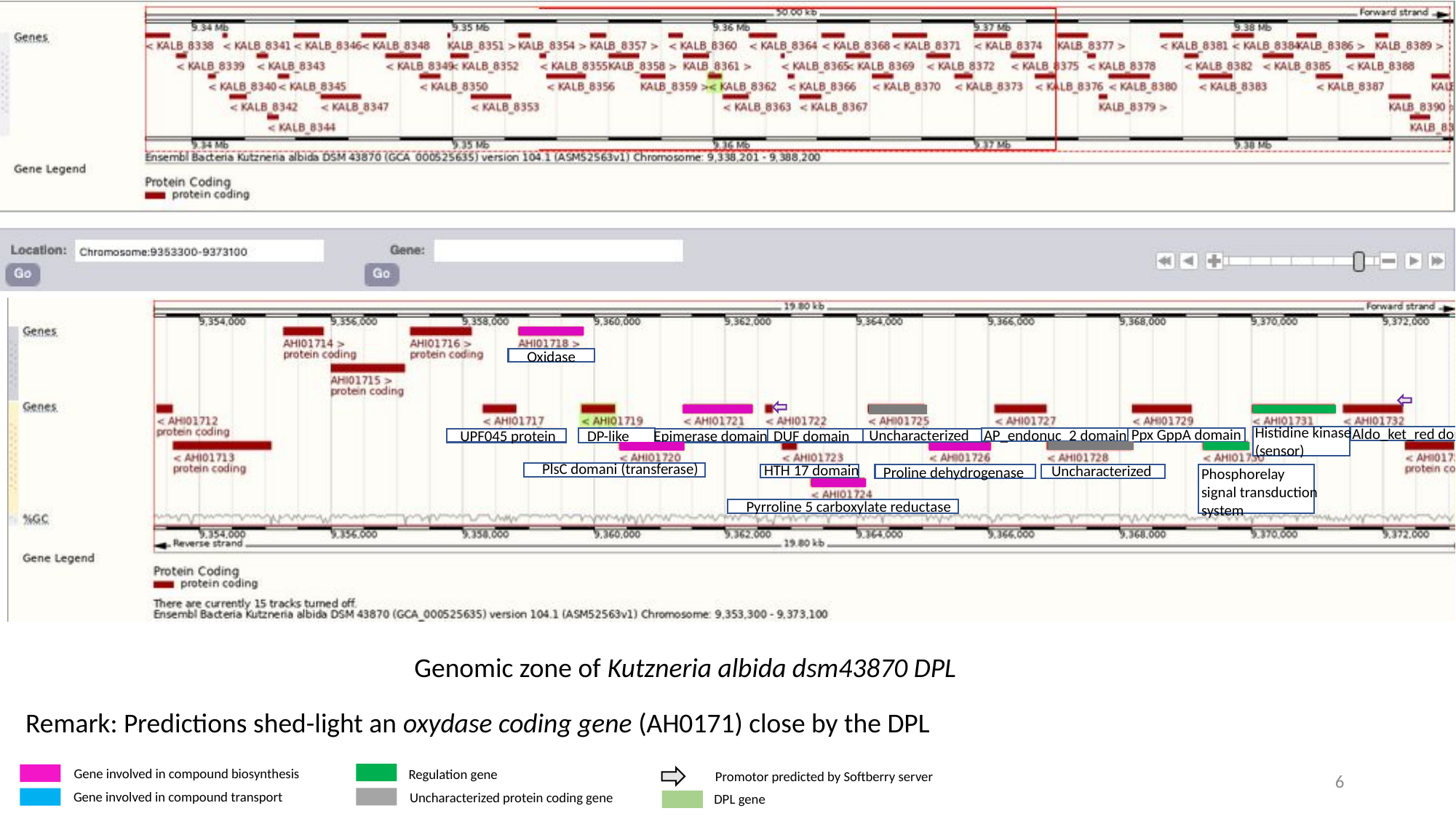

Oxidase
Histidine kinase
(sensor)
Aldo_ket_red dom.
Ppx GppA domain
AP_endonuc_2 domain
Uncharacterized
UPF045 protein
DP-like
Epimerase domain
DUF domain
PlsC domani (transferase)
HTH 17 domain
Uncharacterized
Proline dehydrogenase
Phosphorelay
signal transduction
system
Pyrroline 5 carboxylate reductase
Genomic zone of Kutzneria albida dsm43870 DPL
Remark: Predictions shed-light an oxydase coding gene (AH0171) close by the DPL
6
Gene involved in compound biosynthesis
Regulation gene
Promotor predicted by Softberry server
Gene involved in compound transport
Uncharacterized protein coding gene
DPL gene

## Slide 7
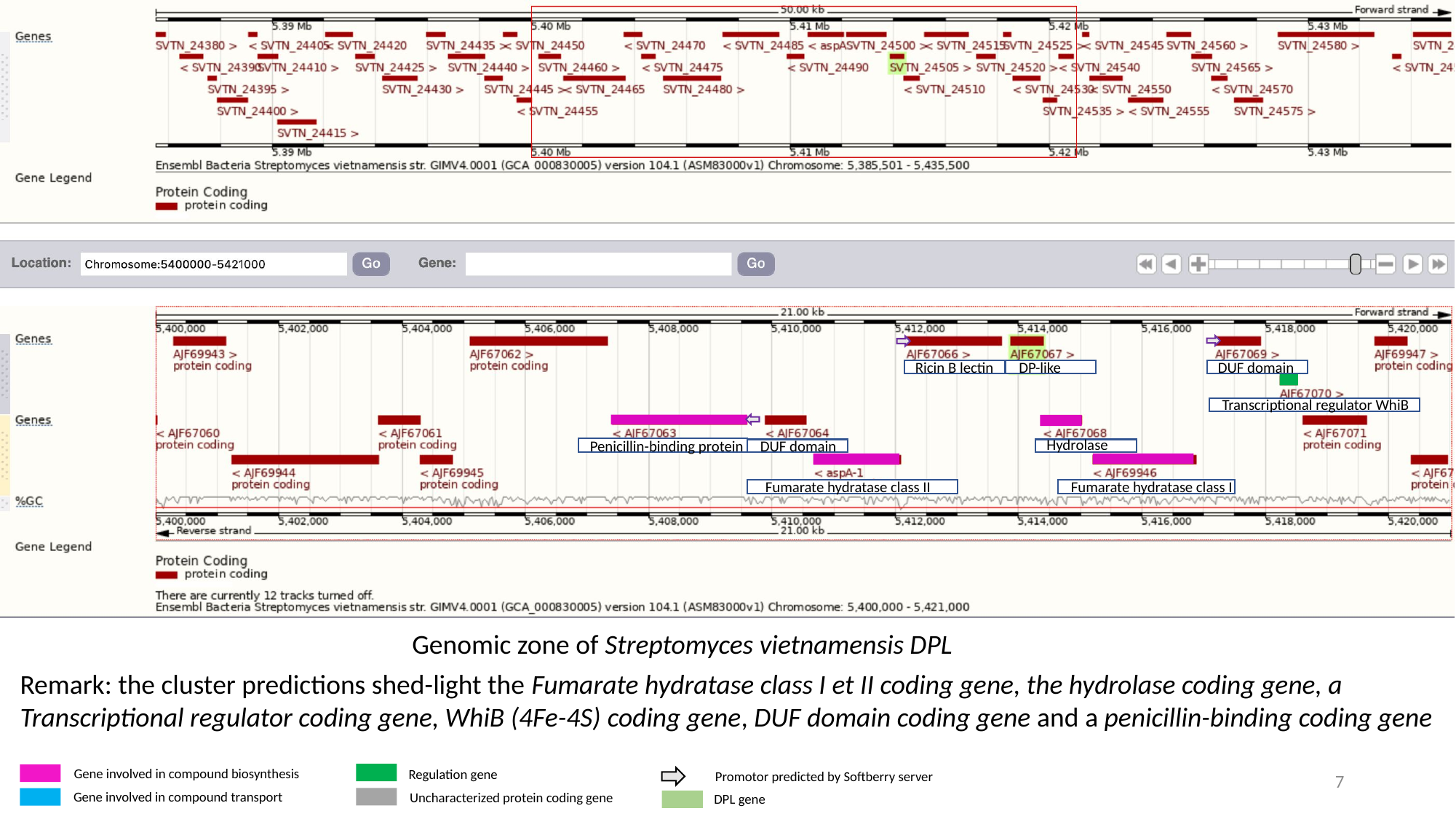

Ricin B lectin
DP-like
DUF domain
Transcriptional regulator WhiB
Hydrolase
Penicillin-binding protein
DUF domain
Fumarate hydratase class II
Fumarate hydratase class I
Genomic zone of Streptomyces vietnamensis DPL
Remark: the cluster predictions shed-light the Fumarate hydratase class I et II coding gene, the hydrolase coding gene, a Transcriptional regulator coding gene, WhiB (4Fe-4S) coding gene, DUF domain coding gene and a penicillin-binding coding gene
7
Gene involved in compound biosynthesis
Regulation gene
Promotor predicted by Softberry server
Gene involved in compound transport
Uncharacterized protein coding gene
DPL gene

## Slide 8
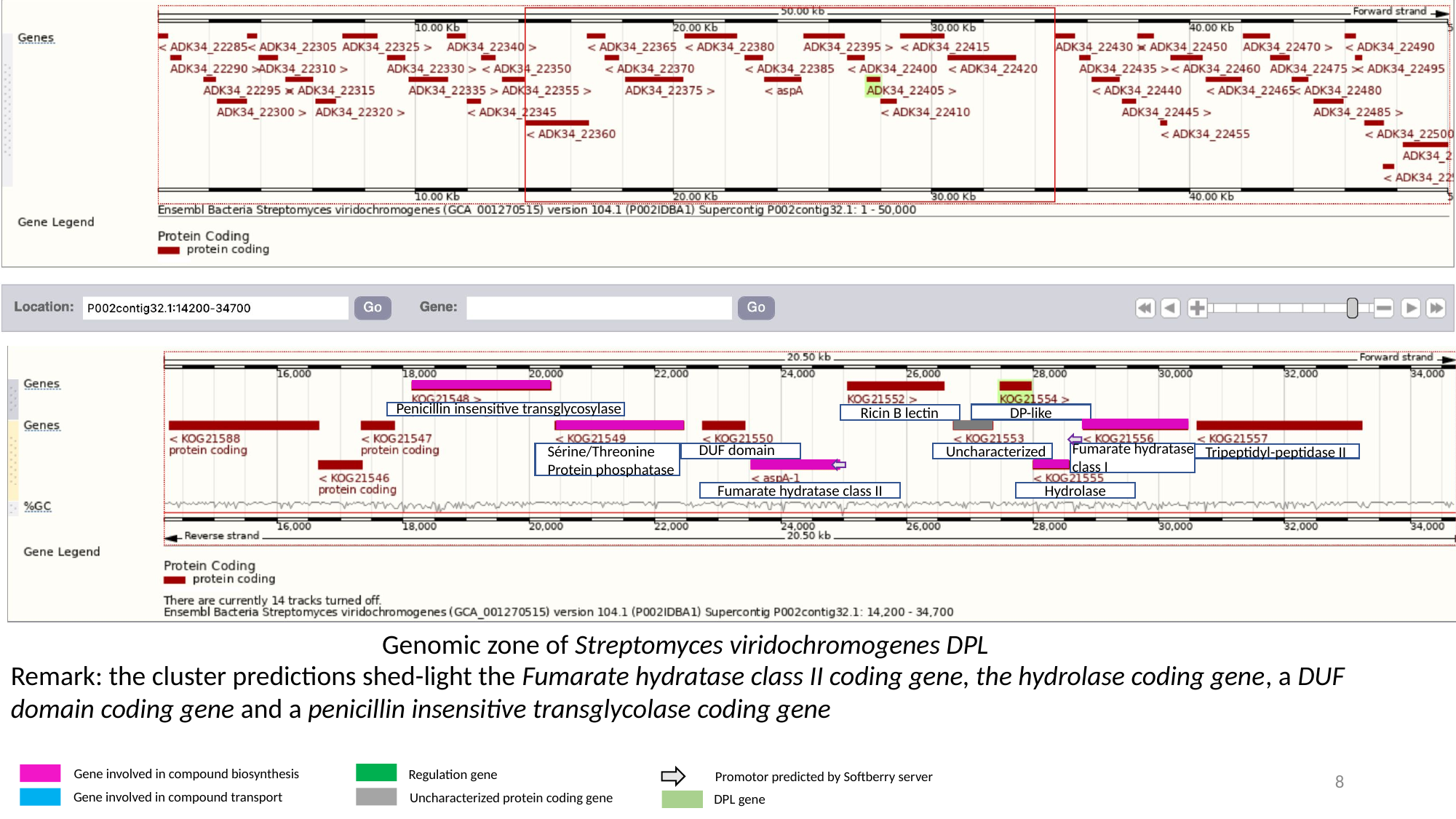

Penicillin insensitive transglycosylase
Ricin B lectin
DP-like
Fumarate hydratase
class I
DUF domain
Sérine/Threonine
Protein phosphatase
Uncharacterized
Tripeptidyl-peptidase II
Fumarate hydratase class II
Hydrolase
Genomic zone of Streptomyces viridochromogenes DPL
Remark: the cluster predictions shed-light the Fumarate hydratase class II coding gene, the hydrolase coding gene, a DUF domain coding gene and a penicillin insensitive transglycolase coding gene
8
Gene involved in compound biosynthesis
Regulation gene
Promotor predicted by Softberry server
Gene involved in compound transport
Uncharacterized protein coding gene
DPL gene

## Slide 9
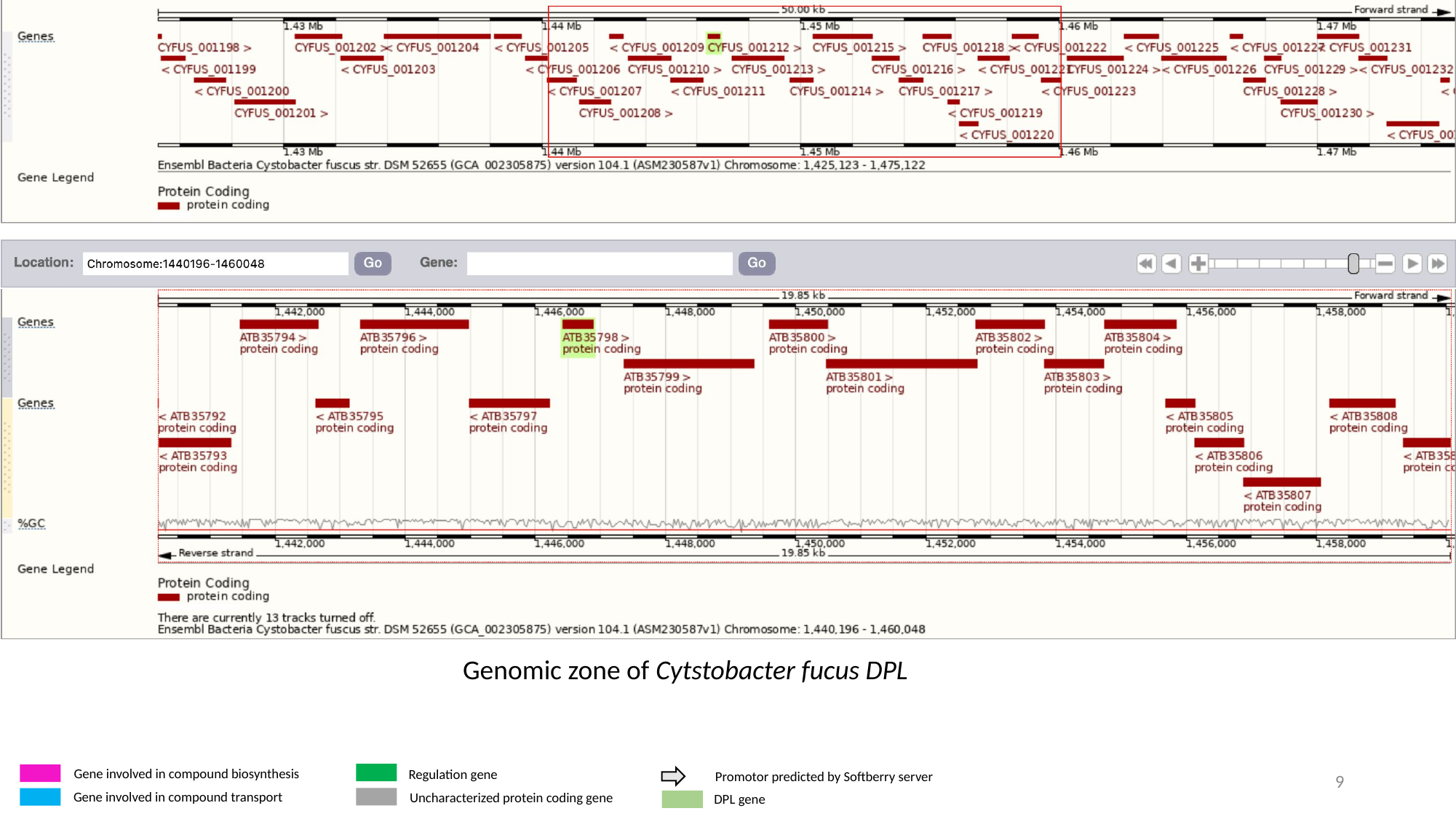

Genomic zone of Cytstobacter fucus DPL
9
Gene involved in compound biosynthesis
Regulation gene
Promotor predicted by Softberry server
Gene involved in compound transport
Uncharacterized protein coding gene
DPL gene

## Slide 10
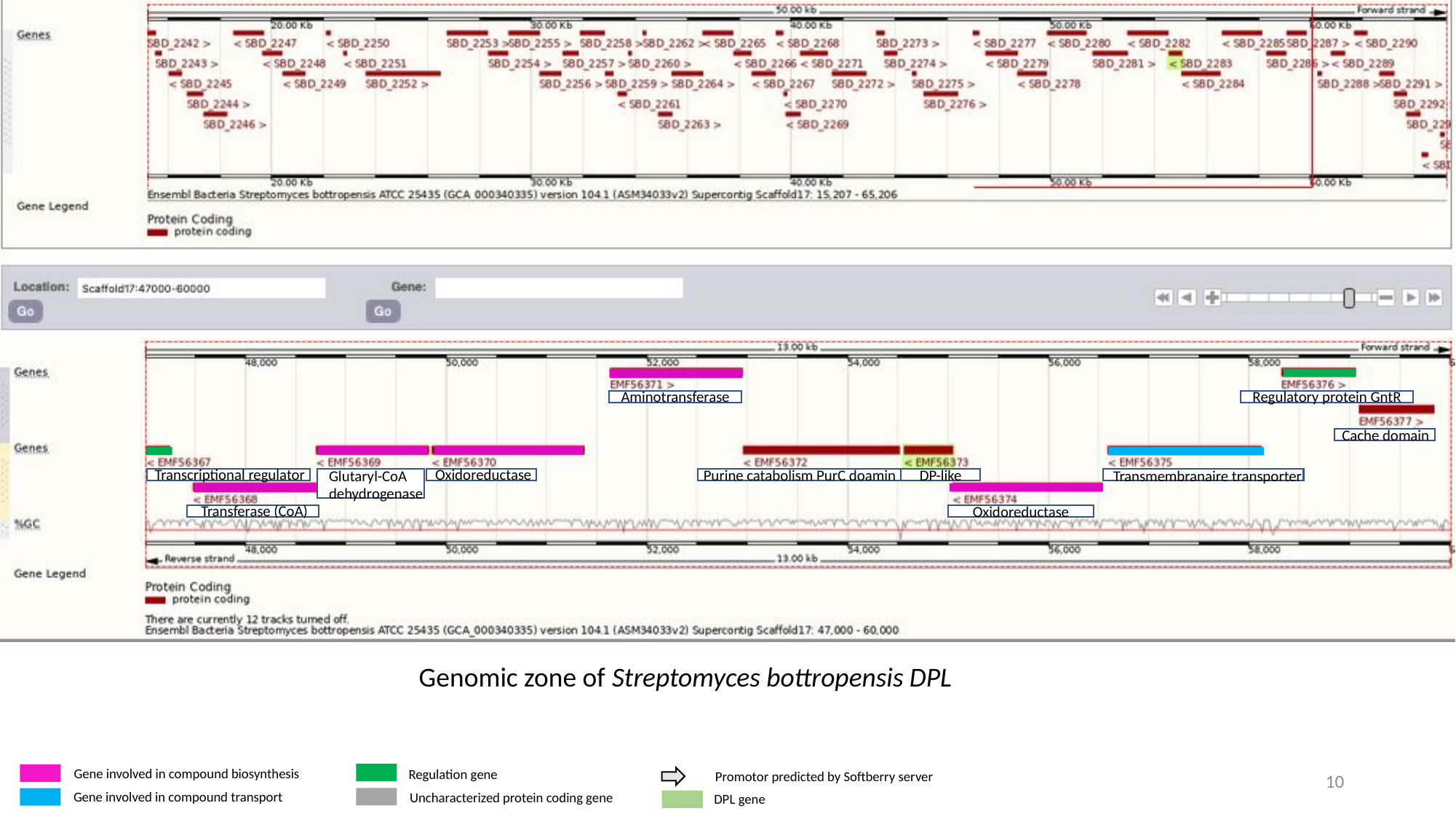

Aminotransferase
Regulatory protein GntR
Cache domain
Transcriptional regulator
Oxidoreductase
Purine catabolism PurC doamin
DP-like
Glutaryl-CoA
dehydrogenase
Transmembranaire transporter
Transferase (CoA)
Oxidoreductase
Genomic zone of Streptomyces bottropensis DPL
10
Gene involved in compound biosynthesis
Regulation gene
Promotor predicted by Softberry server
Gene involved in compound transport
Uncharacterized protein coding gene
DPL gene

## Slide 11
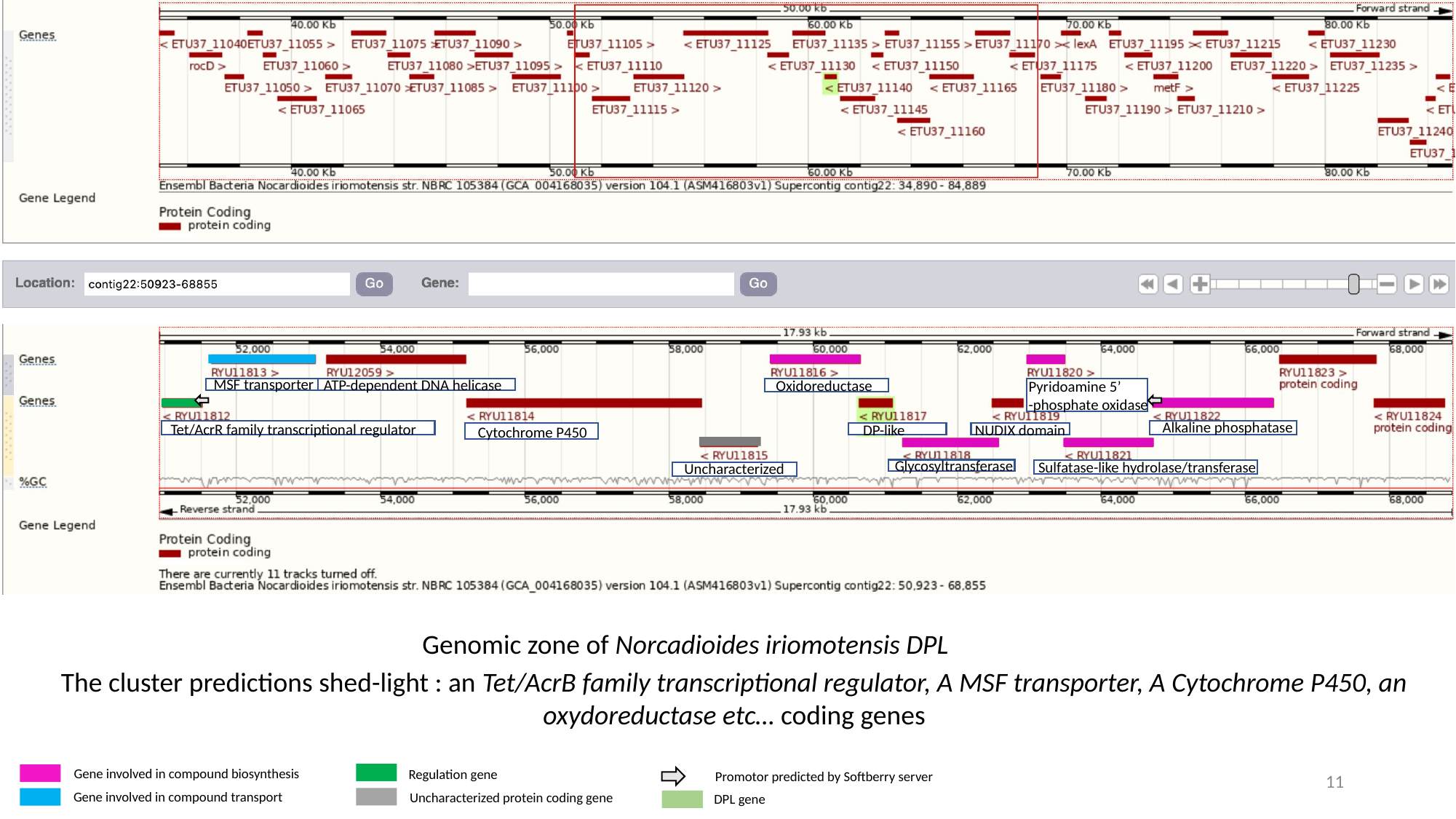

MSF transporter
ATP-dependent DNA helicase
Oxidoreductase
Pyridoamine 5’
-phosphate oxidase
Alkaline phosphatase
Tet/AcrR family transcriptional regulator
NUDIX domain
DP-like
Cytochrome P450
Glycosyltransferase
Sulfatase-like hydrolase/transferase
Uncharacterized
Genomic zone of Norcadioides iriomotensis DPL
The cluster predictions shed-light : an Tet/AcrB family transcriptional regulator, A MSF transporter, A Cytochrome P450, an oxydoreductase etc… coding genes
11
Gene involved in compound biosynthesis
Regulation gene
Promotor predicted by Softberry server
Gene involved in compound transport
Uncharacterized protein coding gene
DPL gene

## Slide 12
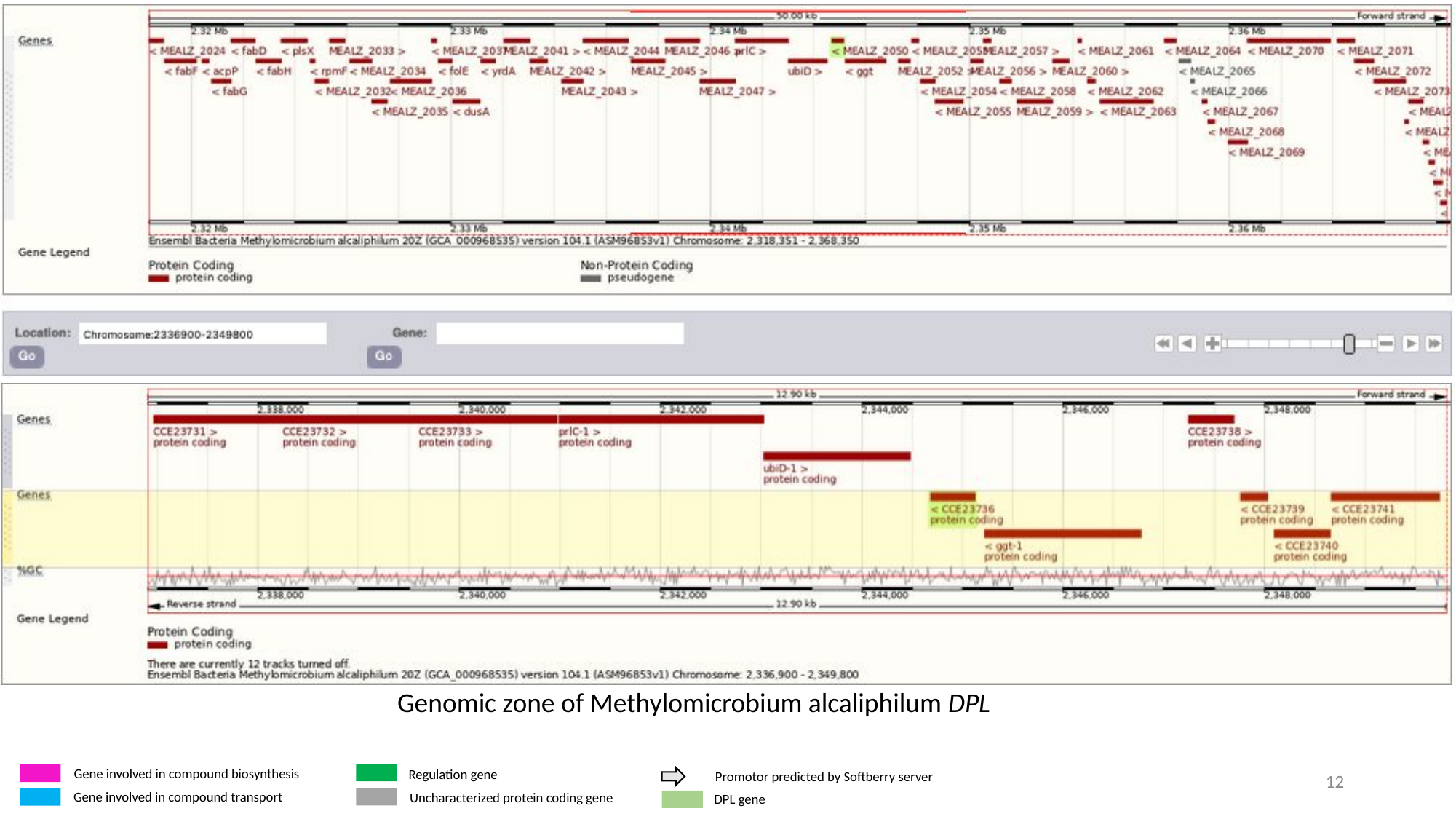

Genomic zone of Methylomicrobium alcaliphilum DPL
12
Gene involved in compound biosynthesis
Regulation gene
Promotor predicted by Softberry server
Gene involved in compound transport
Uncharacterized protein coding gene
DPL gene

## Slide 13
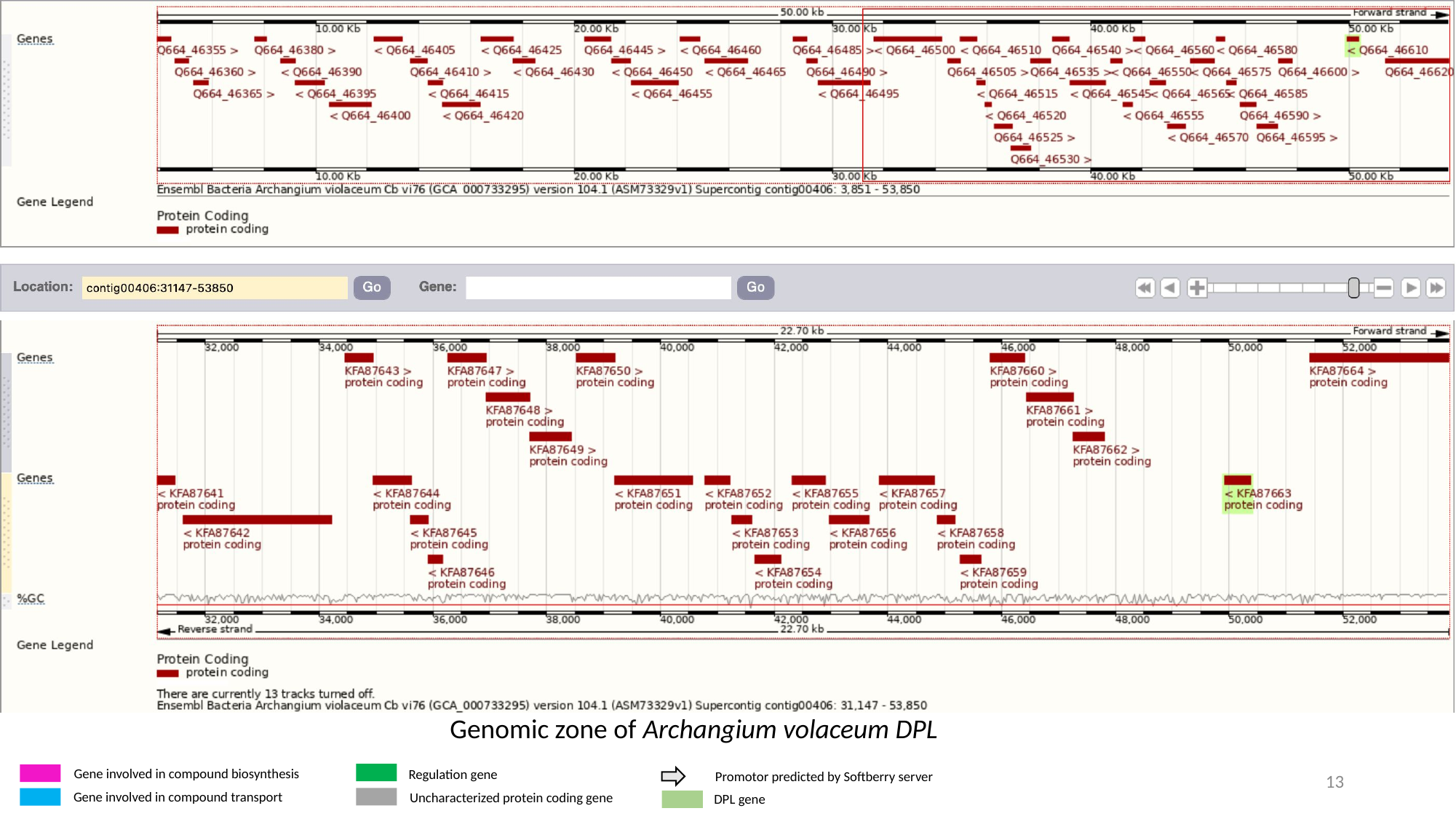

Genomic zone of Archangium volaceum DPL
13
Gene involved in compound biosynthesis
Regulation gene
Promotor predicted by Softberry server
Gene involved in compound transport
Uncharacterized protein coding gene
DPL gene

## Slide 14
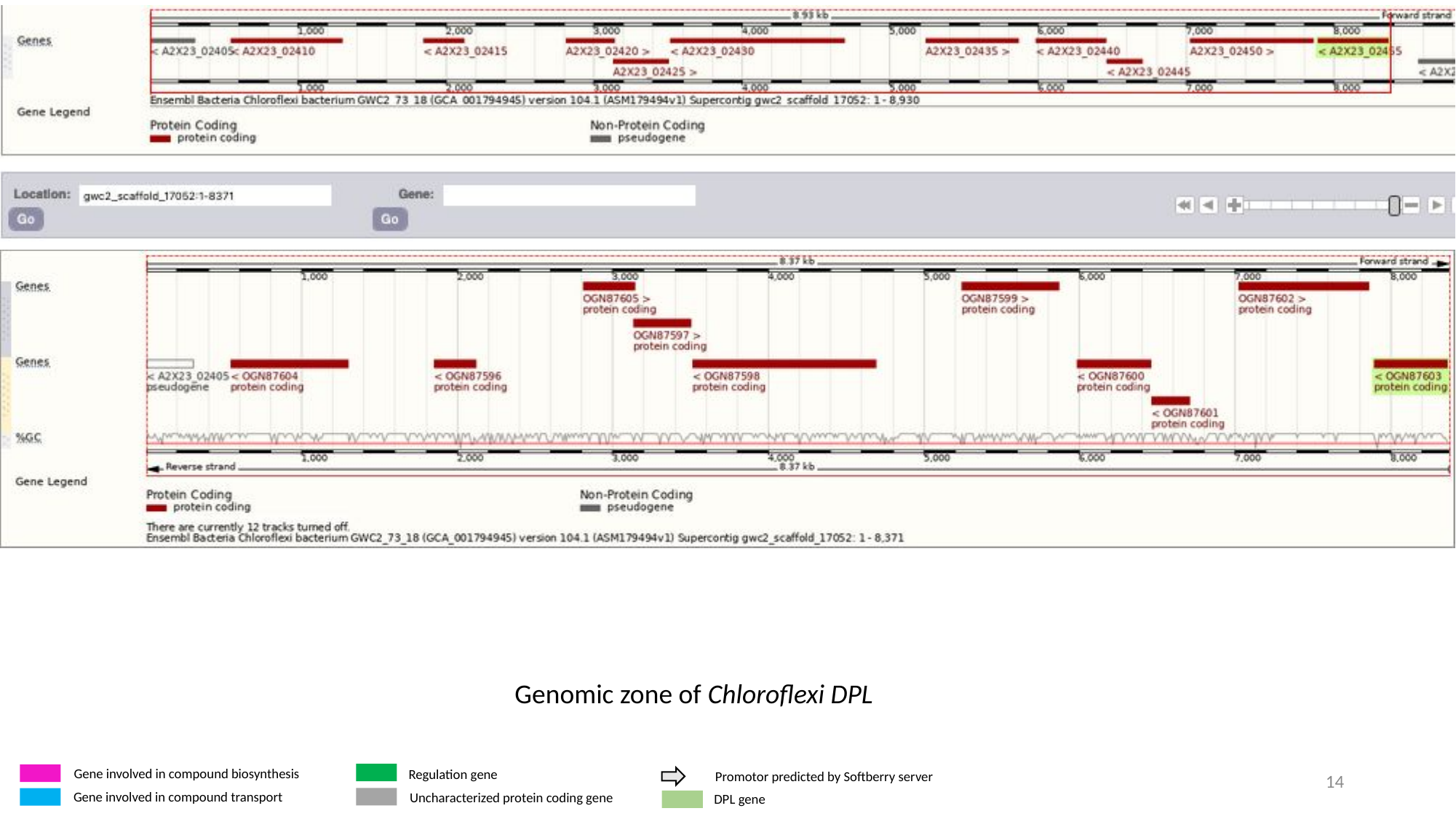

Genomic zone of Chloroflexi DPL
14
Gene involved in compound biosynthesis
Regulation gene
Promotor predicted by Softberry server
Gene involved in compound transport
Uncharacterized protein coding gene
DPL gene

## Slide 15
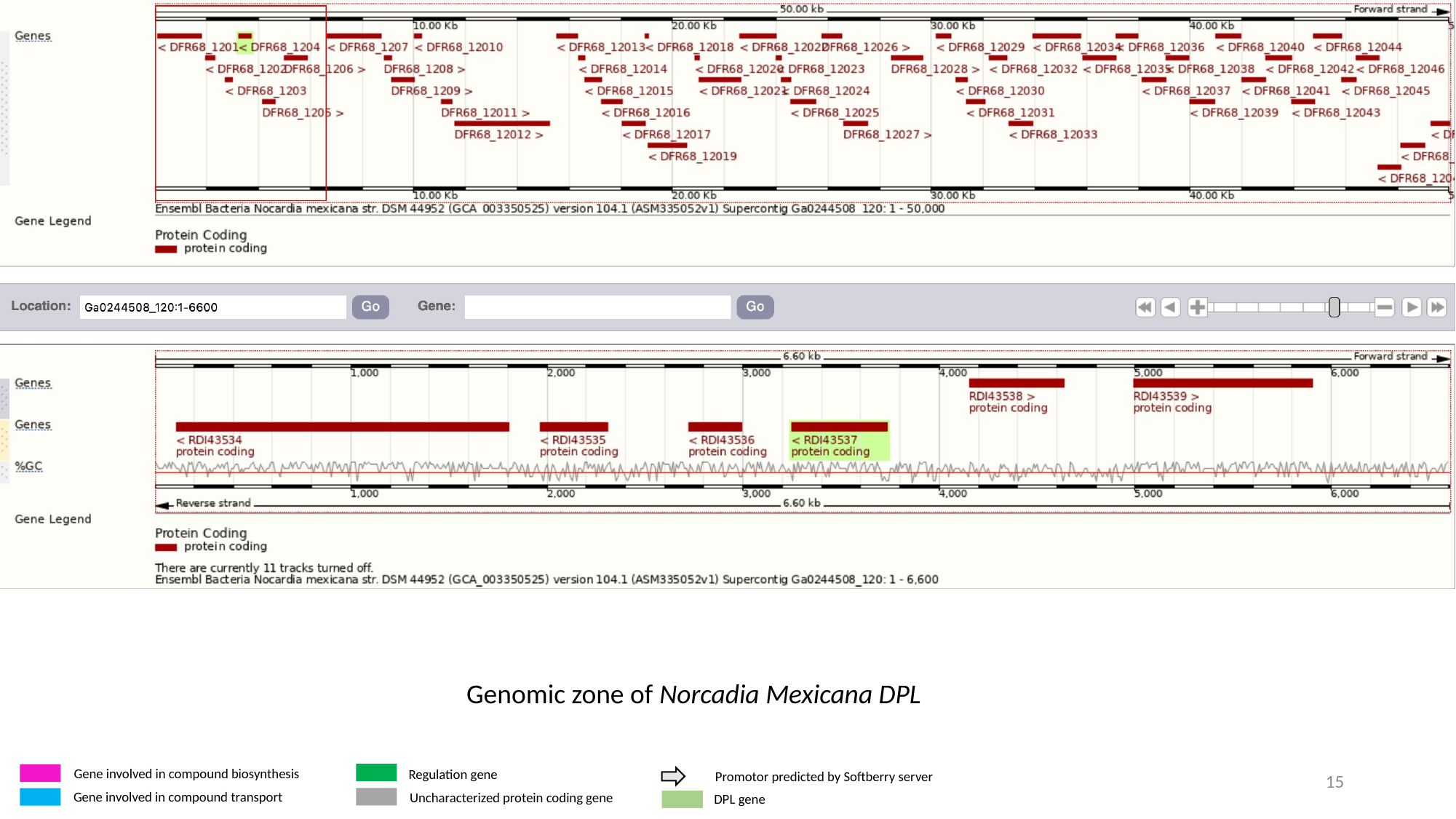

Genomic zone of Norcadia Mexicana DPL
15
Gene involved in compound biosynthesis
Regulation gene
Promotor predicted by Softberry server
Gene involved in compound transport
Uncharacterized protein coding gene
DPL gene

## Slide 16
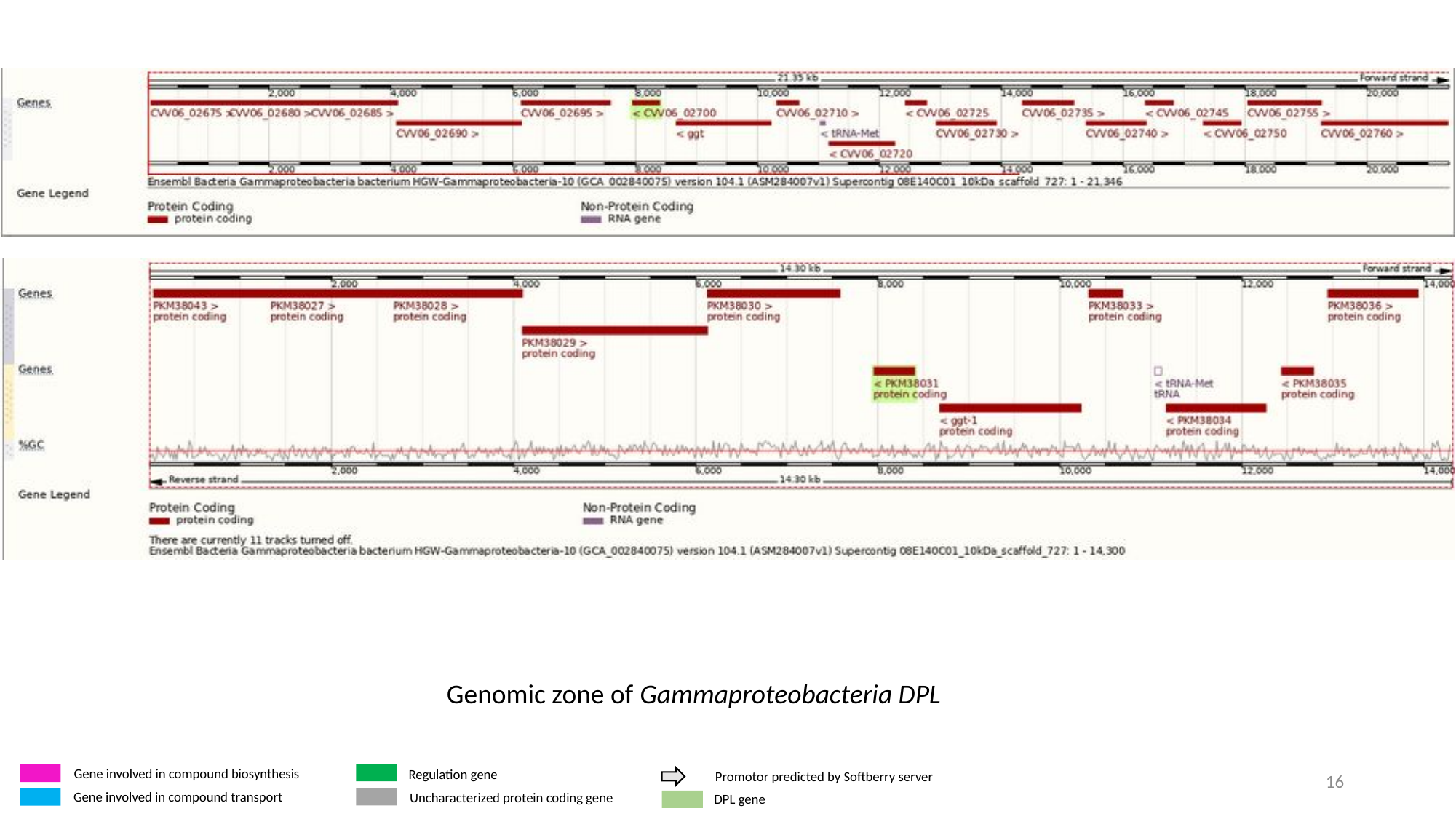

Genomic zone of Gammaproteobacteria DPL
16
Gene involved in compound biosynthesis
Regulation gene
Promotor predicted by Softberry server
Gene involved in compound transport
Uncharacterized protein coding gene
DPL gene

## Slide 17
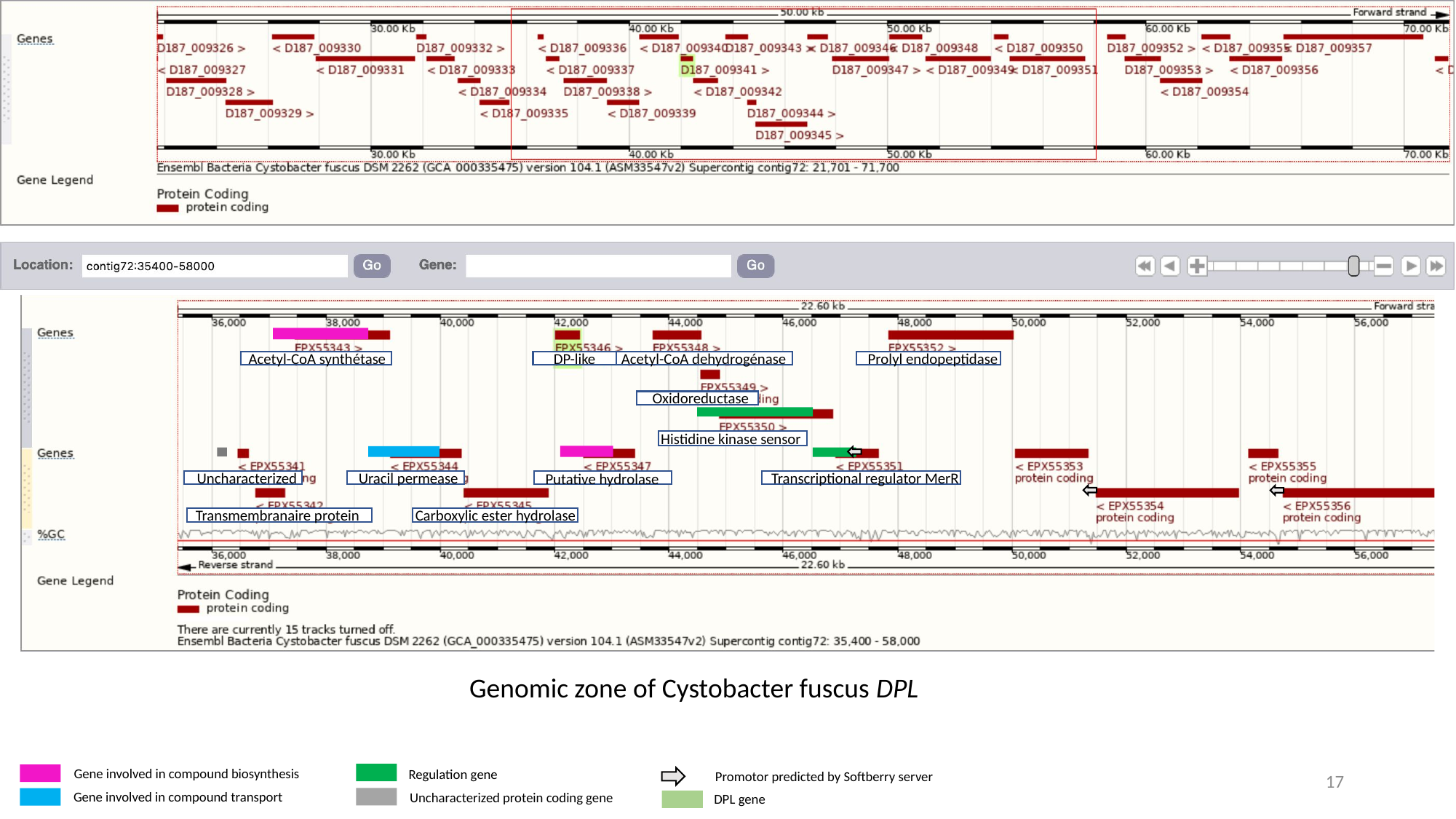

Prolyl endopeptidase
Acetyl-CoA synthétase
Acetyl-CoA dehydrogénase
DP-like
Oxidoreductase
Histidine kinase sensor
Uncharacterized
Uracil permease
Transcriptional regulator MerR
Putative hydrolase
Transmembranaire protein
Carboxylic ester hydrolase
Genomic zone of Cystobacter fuscus DPL
17
Gene involved in compound biosynthesis
Regulation gene
Promotor predicted by Softberry server
Gene involved in compound transport
Uncharacterized protein coding gene
DPL gene

## Slide 18
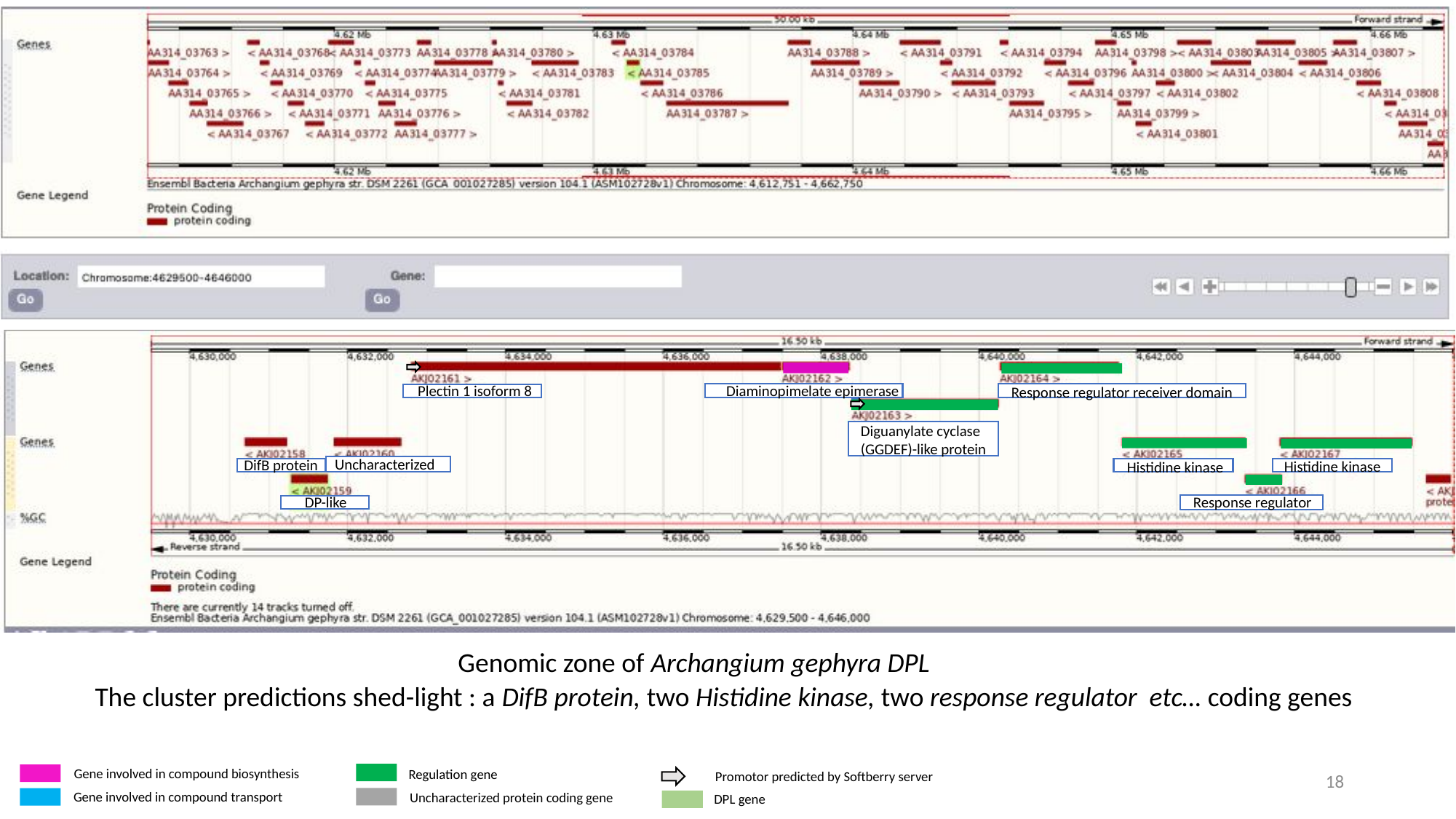

Plectin 1 isoform 8
Diaminopimelate epimerase
Response regulator receiver domain
Diguanylate cyclase
(GGDEF)-like protein
Uncharacterized
DifB protein
Histidine kinase
Histidine kinase
DP-like
Response regulator
Genomic zone of Archangium gephyra DPL
The cluster predictions shed-light : a DifB protein, two Histidine kinase, two response regulator etc… coding genes
18
Gene involved in compound biosynthesis
Regulation gene
Promotor predicted by Softberry server
Gene involved in compound transport
Uncharacterized protein coding gene
DPL gene

## Slide 19
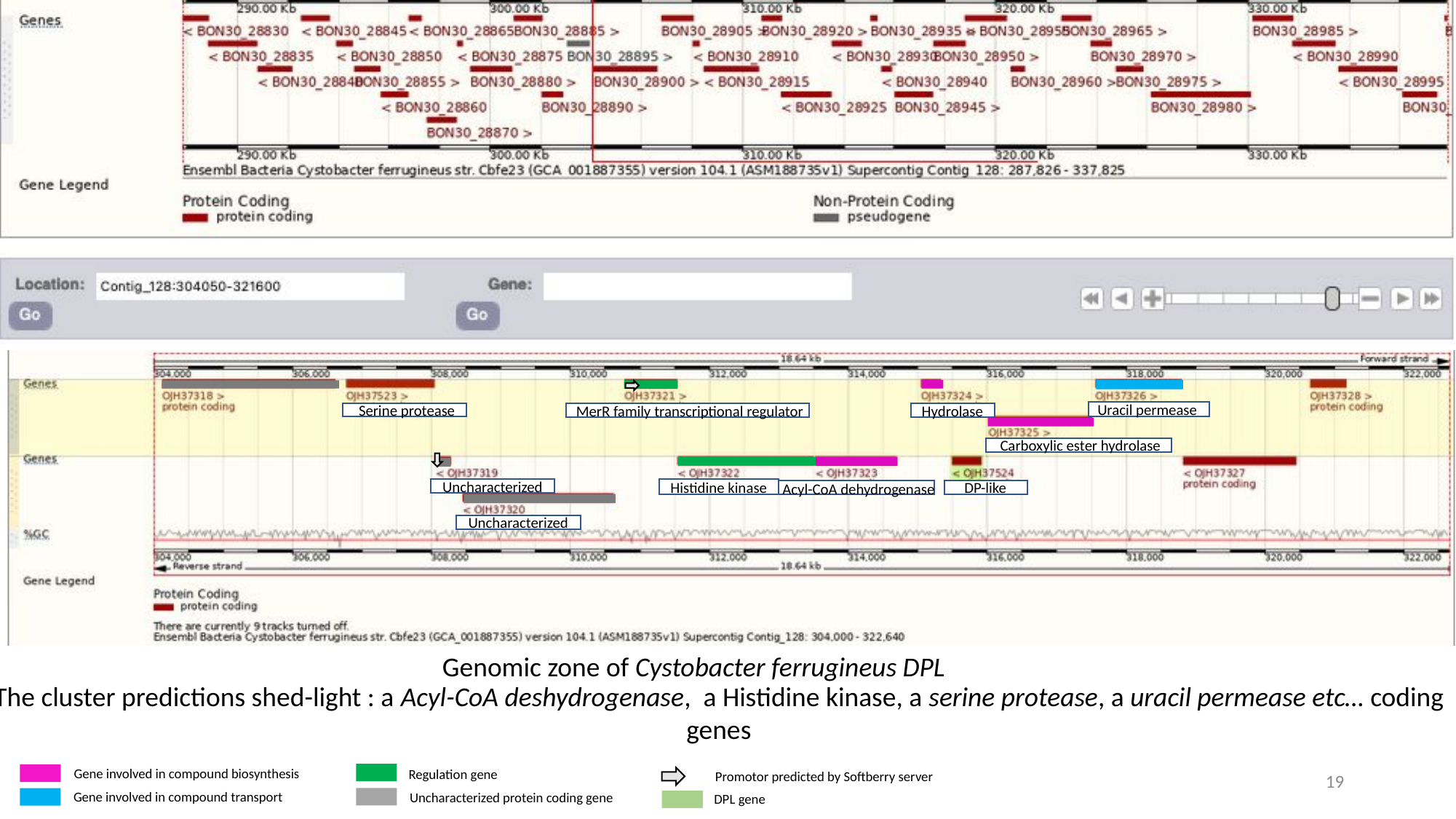

Uracil permease
Serine protease
Hydrolase
MerR family transcriptional regulator
Carboxylic ester hydrolase
Uncharacterized
Histidine kinase
DP-like
Acyl-CoA dehydrogenase
Uncharacterized
Genomic zone of Cystobacter ferrugineus DPL
The cluster predictions shed-light : a Acyl-CoA deshydrogenase, a Histidine kinase, a serine protease, a uracil permease etc… coding genes
19
Gene involved in compound biosynthesis
Regulation gene
Promotor predicted by Softberry server
Gene involved in compound transport
Uncharacterized protein coding gene
DPL gene

## Slide 20
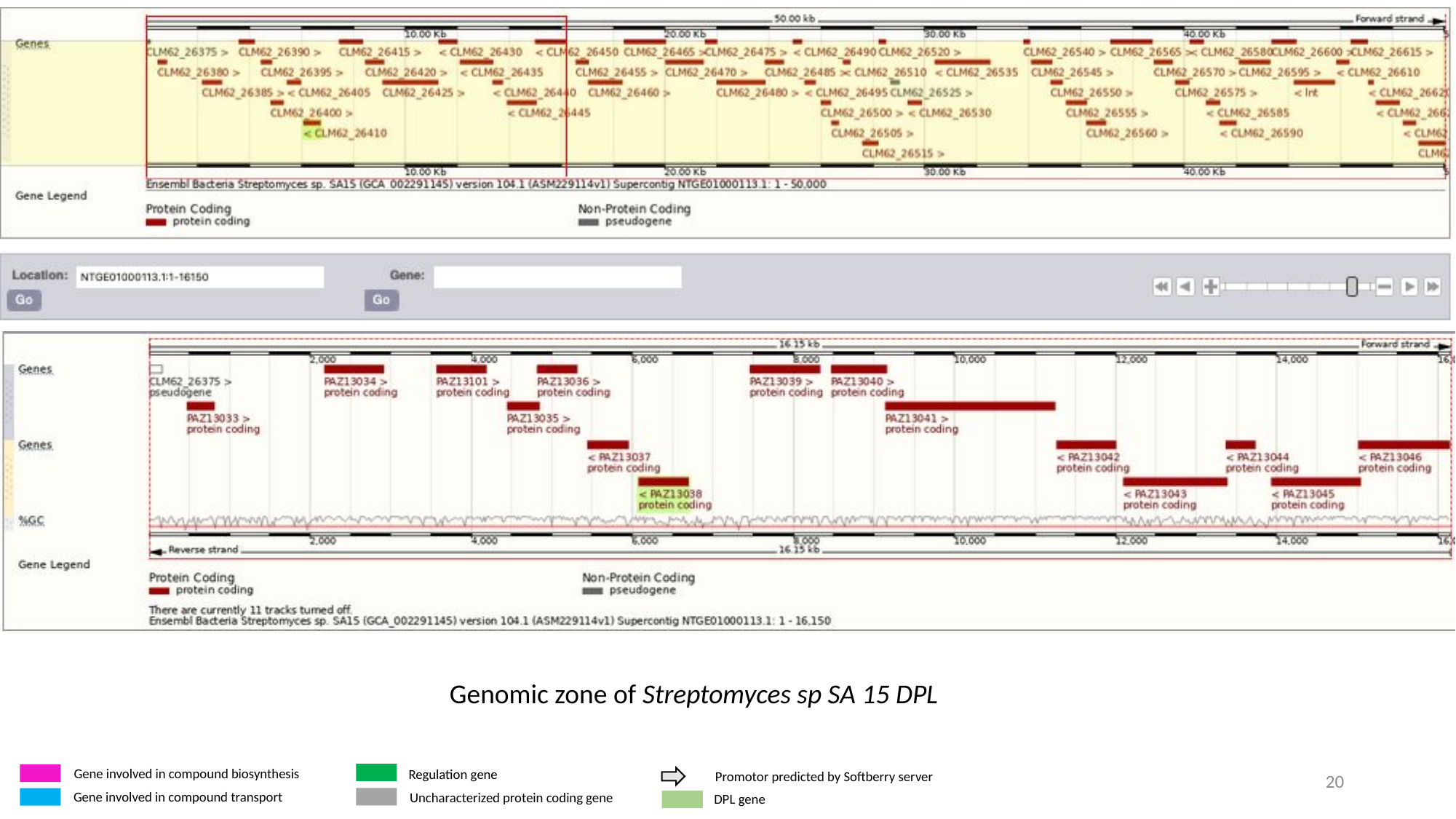

Genomic zone of Streptomyces sp SA 15 DPL
20
Gene involved in compound biosynthesis
Regulation gene
Promotor predicted by Softberry server
Gene involved in compound transport
Uncharacterized protein coding gene
DPL gene

## Slide 21
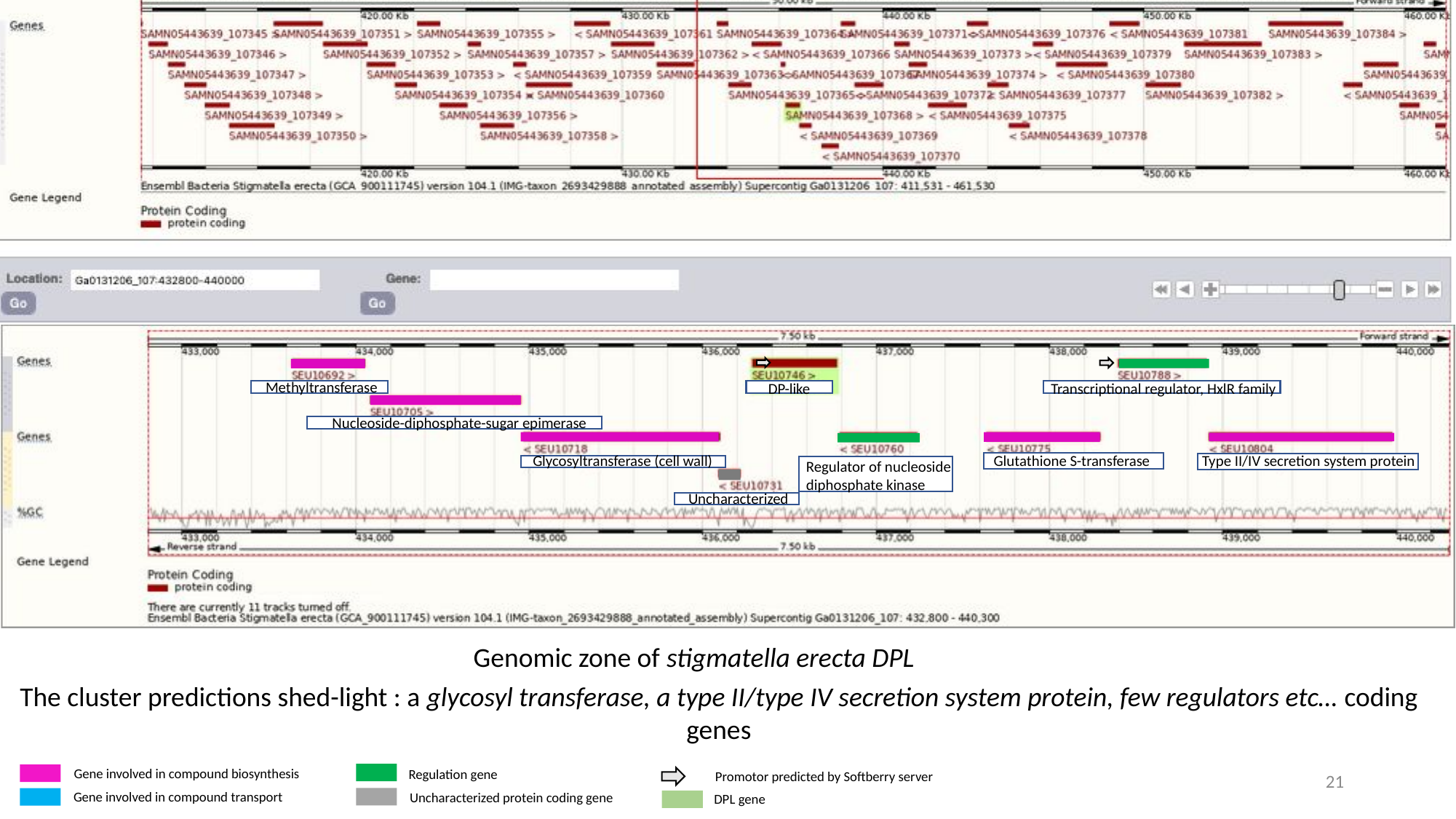

Methyltransferase
DP-like
Transcriptional regulator, HxlR family
Nucleoside-diphosphate-sugar epimerase
Glutathione S-transferase
Glycosyltransferase (cell wall)
Type II/IV secretion system protein
Regulator of nucleoside
diphosphate kinase
Uncharacterized
Genomic zone of stigmatella erecta DPL
The cluster predictions shed-light : a glycosyl transferase, a type II/type IV secretion system protein, few regulators etc… coding genes
21
Gene involved in compound biosynthesis
Regulation gene
Promotor predicted by Softberry server
Gene involved in compound transport
Uncharacterized protein coding gene
DPL gene

## Slide 22
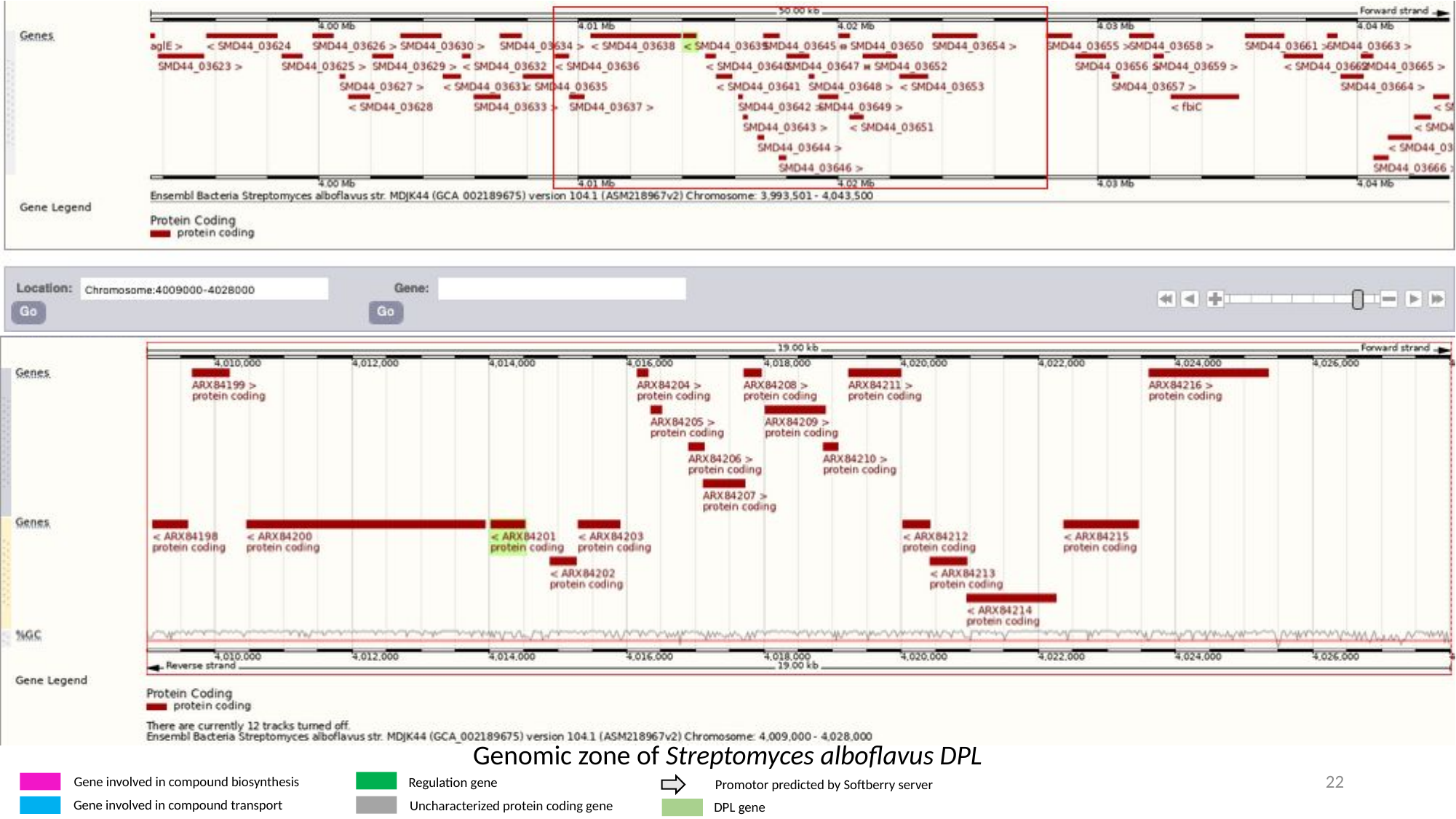

Genomic zone of Streptomyces alboflavus DPL
22
Gene involved in compound biosynthesis
Regulation gene
Promotor predicted by Softberry server
Gene involved in compound transport
Uncharacterized protein coding gene
DPL gene

## Slide 23
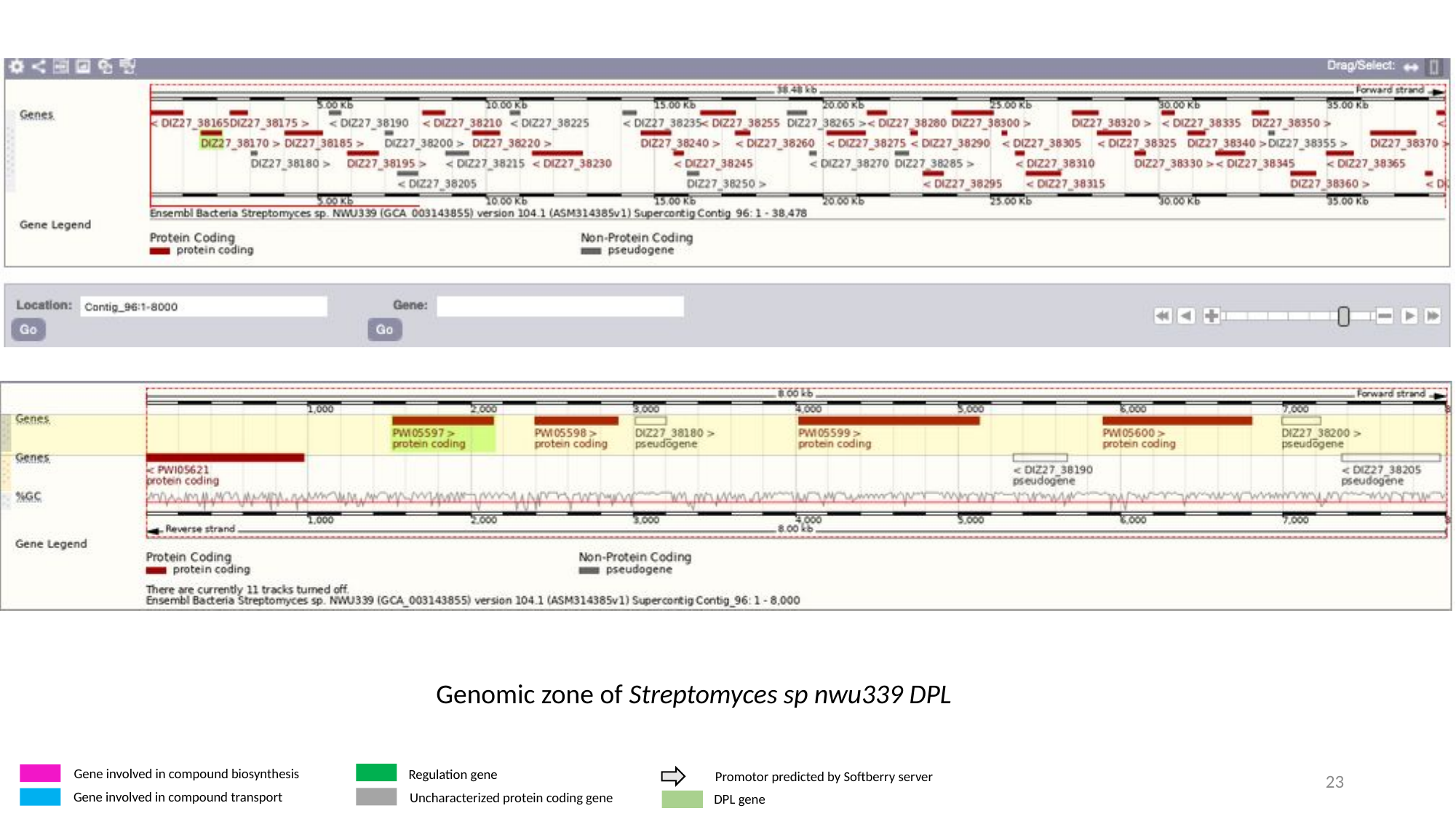

Genomic zone of Streptomyces sp nwu339 DPL
23
Gene involved in compound biosynthesis
Regulation gene
Promotor predicted by Softberry server
Gene involved in compound transport
Uncharacterized protein coding gene
DPL gene

## Slide 24
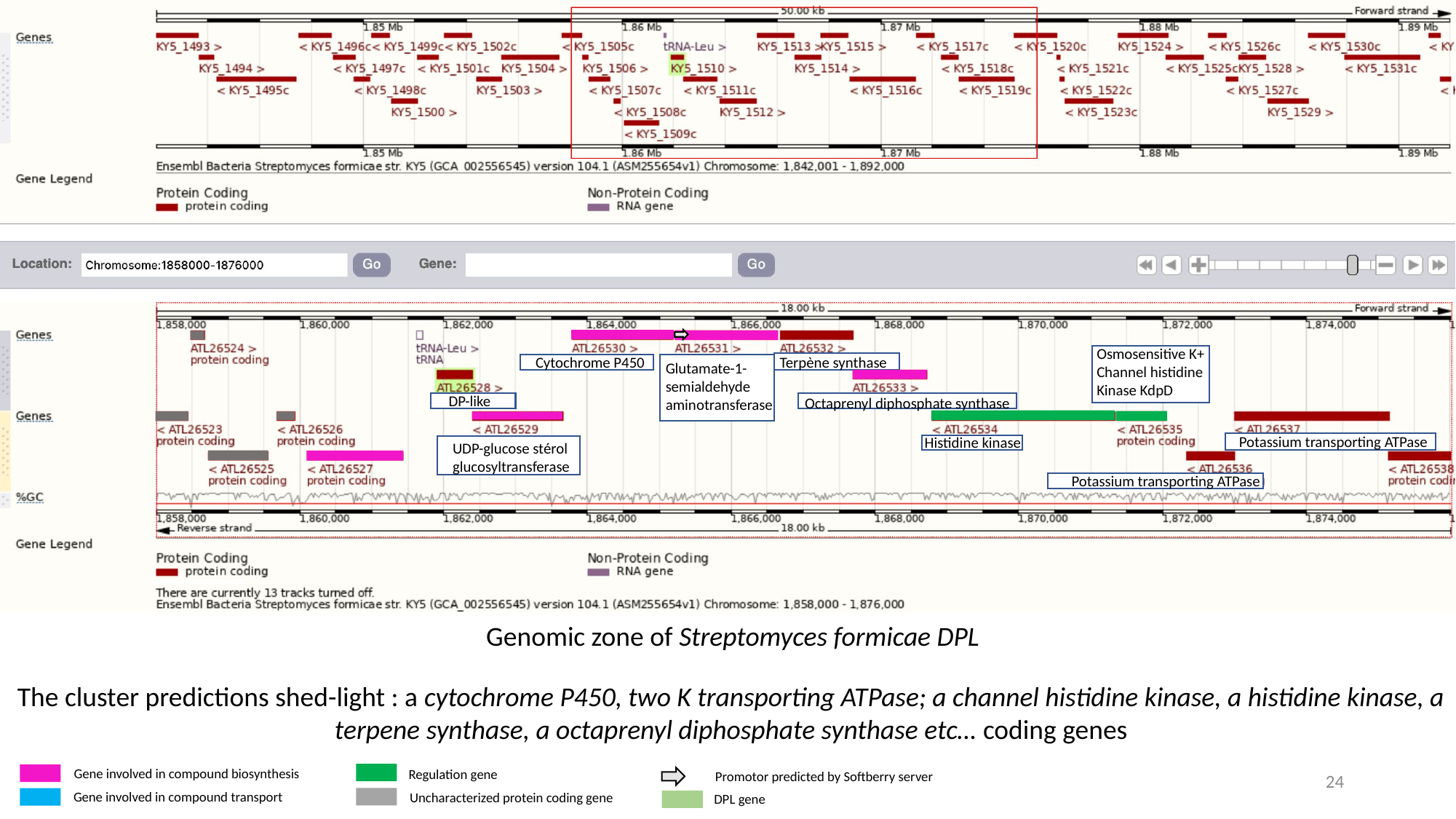

Osmosensitive K+
Channel histidine
Kinase KdpD
Cytochrome P450
Terpène synthase
Glutamate-1-
semialdehyde
aminotransferase
DP-like
Octaprenyl diphosphate synthase
Potassium transporting ATPase
Histidine kinase
UDP-glucose stérol
glucosyltransferase
Potassium transporting ATPase
Genomic zone of Streptomyces formicae DPL
The cluster predictions shed-light : a cytochrome P450, two K transporting ATPase; a channel histidine kinase, a histidine kinase, a terpene synthase, a octaprenyl diphosphate synthase etc… coding genes
24
Gene involved in compound biosynthesis
Regulation gene
Promotor predicted by Softberry server
Gene involved in compound transport
Uncharacterized protein coding gene
DPL gene

## Slide 25
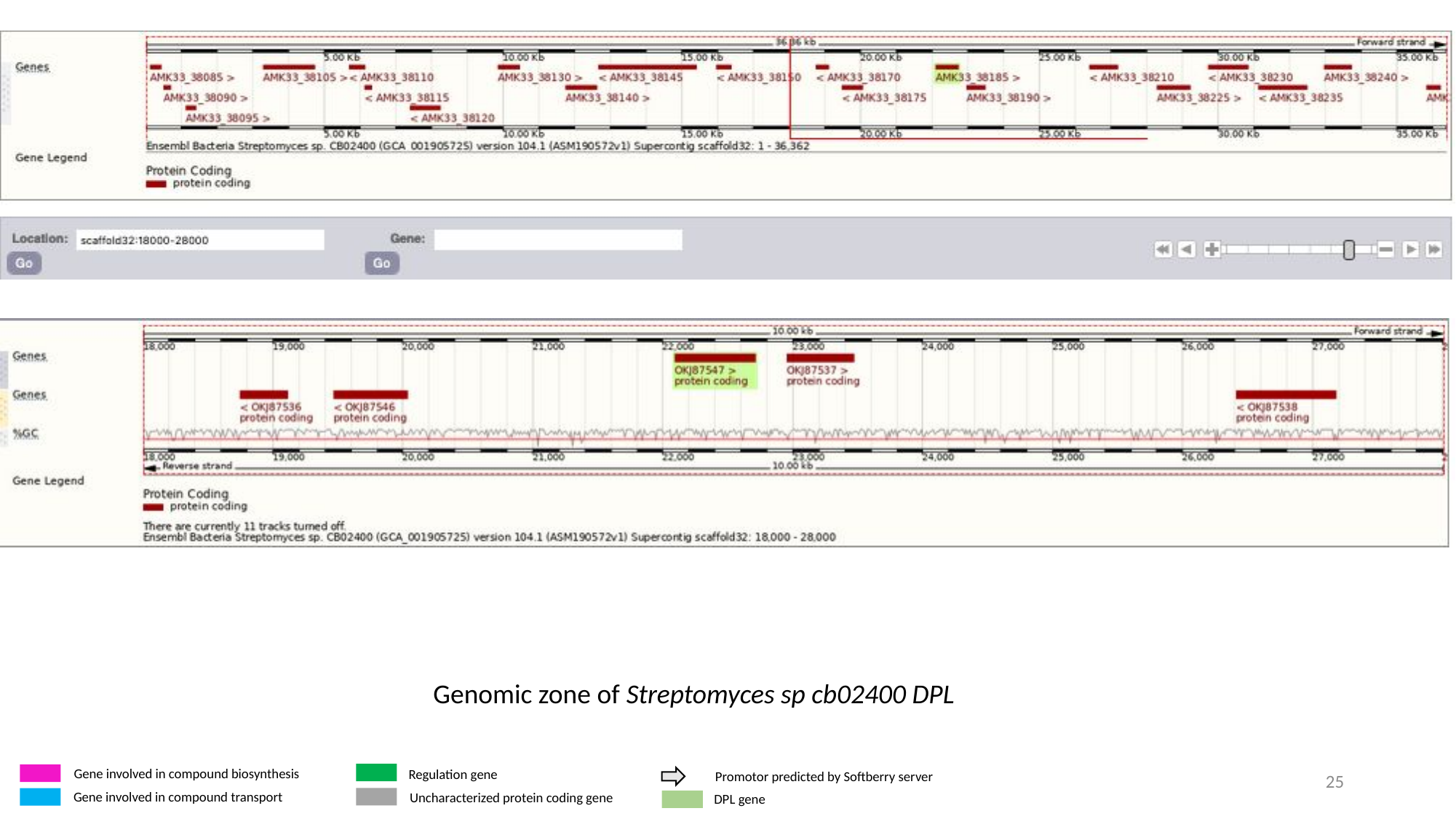

Genomic zone of Streptomyces sp cb02400 DPL
25
Gene involved in compound biosynthesis
Regulation gene
Promotor predicted by Softberry server
Gene involved in compound transport
Uncharacterized protein coding gene
DPL gene

## Slide 26
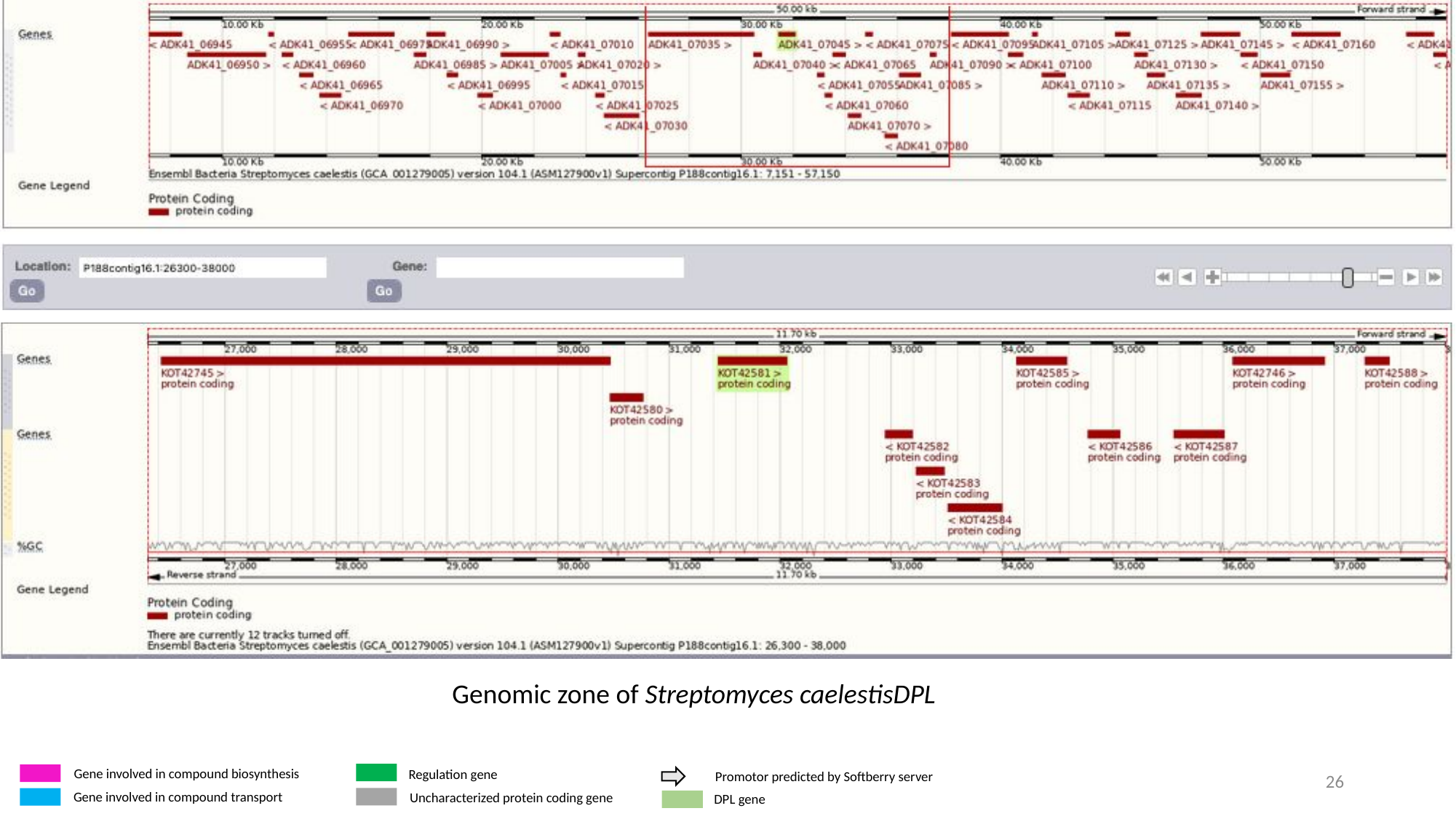

Genomic zone of Streptomyces caelestisDPL
26
Gene involved in compound biosynthesis
Regulation gene
Promotor predicted by Softberry server
Gene involved in compound transport
Uncharacterized protein coding gene
DPL gene
